# Cellular processes of v-Src transformation revealed by gene profiling of primary cells - Implications for human cancer

**DOI:** 10.1186/1471-2407-10-41

**Published:** 2010-02-12

**Authors:** Bart M Maślikowski, Benjamin D Néel, Ying Wu, Lizhen Wang, Natalie A Rodrigues, Germain Gillet, Pierre-André Bédard

**Affiliations:** 1 Department of Biology, McMaster University, 1280 Main street West, Hamilton, ON, L8S 4K1, Canada; 2 Institut de Biologie et Chimie des Protéines, Lyon, France; 3 Sunnybrook Health Sciences Centre, 2075 Bayview Avenue, Toronto, ON, M4N 3 M5, Canada

## Abstract

**Background:**

Cell transformation by the Src tyrosine kinase is characterized by extensive changes in gene expression. In this study, we took advantage of several strains of the Rous sarcoma virus (RSV) to characterize the patterns of v-Src-dependent gene expression in two different primary cell types, namely chicken embryo fibroblasts (CEF) and chicken neuroretinal (CNR) cells. We identified a common set of v-Src regulated genes and assessed if their expression is associated with disease-free survival using several independent human tumor data sets.

**Methods:**

CEF and CNR cells were infected with transforming, non-transforming, and temperature sensitive mutants of RSV to identify the patterns of gene expression in response to v-Src-transformation. Microarray analysis was used to measure changes in gene expression and to define a common set of v-Src regulated genes (CSR genes) in CEF and CNR cells. A clustering enrichment regime using the CSR genes and two independent breast tumor data-sets was used to identify a 42-gene aggressive tumor gene signature. The aggressive gene signature was tested for its prognostic value by conducting survival analyses on six additional tumor data sets.

**Results:**

The analysis of CEF and CNR cells revealed that cell transformation by v-Src alters the expression of 6% of the protein coding genes of the genome. A common set of 175 v-Src regulated genes (CSR genes) was regulated in both CEF and CNR cells. Within the CSR gene set, a group of 42 v-Src inducible genes was associated with reduced disease- and metastasis-free survival in several independent patient cohorts with breast or lung cancer. Gene classes represented within this group include DNA replication, cell cycle, the DNA damage and stress responses, and blood vessel morphogenesis.

**Conclusion:**

By studying the v-Src-dependent changes in gene expression in two types of primary cells, we identified a set of 42 inducible genes associated with poor prognosis in breast and lung cancer. The identification of these genes provides a set of biomarkers of aggressive tumor behavior and a framework for the study of cancer cells characterized by elevated Src kinase activity.

## Background

The v-Src kinase, the product of the Rous sarcoma virus (RSV), has provided a paradigm for the study of signaling pathways and mechanisms of cell transformation by receptor and non-receptor type tyrosine kinases. Its cellular counterpart, c-Src, is a member of a small family of kinases sharing a similar domain organization, overall structure and regulatory mechanism. Members of the Src family of kinases (SFK) contribute to several aspects of the activity of receptor tyrosine kinases including receptor turn-over, reorganization of the cytoskeleton and the initiation of DNA synthesis [1].

A role for c-Src in the initiation or progression of human cancer has been documented in several studies. Elevated Src kinase activity has been observed in several human cancers and in particular in breast, ovary, lung, bladder, stomach and colon carcinomas [2]. The majority of breast tumors samples (>70%) show elevated Src kinase activity that reflects increased protein levels [3]. While c-Src over-expression is not sufficient to induce cell transformation, c-Src likely cooperates with other tyrosine kinases, such as the EGF receptor, frequently over-expressed in the same tumors. An activating mutation resulting in the deletion of the c-Src C-terminal region adjacent to the negative regulatory tyrosine (Y530) has also been identified in a subset of patients with advanced colon carcinomas [4]. This mutation mimics the oncogenic activation of v-Src, whose C-terminus lacks the C-terminal Src kinase (Csk) phosphorylation site.

Signaling pathways controlling cell proliferation or survival, in particular the Ras and PI3K pathways, have been the subject of intense investigation in v-Src transformed cells [5-7]. More recently, elevated Src kinase activity has been linked to several aspects of tumorigenesis including modification of the tumor micro-environment, vasculogenesis, metastasis and the acquisition of chemoresistance [8,9]. The mechanisms by which Src controls these properties of tumor cells remain largely unknown. One of the earliest and defining observations of v-Src transformation is the capacity of this oncoprotein to modify the pattern of gene expression. This was revealed by the cloning and characterization of genes aberrantly expressed in v-Src transformed cells, including genes encoding metalloproteinases and chemokines, and the trans-acting factors regulating their expression [10-14]. Investigations based on gene disruption or the use of dominant-negative mutants established the importance of transcription factors such as AP-1, members of the STAT and Ets families, and c-Myc in the proliferation and behavior of v-Src transformed cells [15-22].

Gene profiling studies of transformed cells or tumors characterized by elevated Src kinase activity have documented the changes in gene expression associated with this oncoprotein [23-26]. However, these studies did not assess the transformation-dependence of gene expression [23]. Often they were performed on heterogeneous tumor specimens that limit the sensitivity of the analysis or in immortalized cell lines that may not reveal the full range of Src-dependent changes in gene expression. In this study, we employ a different strategy by comparing the expression profiles of two different primary cell types infected by the Rous sarcoma virus, namely chicken embryo fibroblast (CEF) and chicken neuroretinal cells (CNR). By using temperature sensitive or transformation deficient mutants of v-Src, we define a common set of genes regulated by v-Src. The expression of a group of 42 v-Src inducible genes of the common set was associated with reduced disease-free survival in independent cohorts of patients with breast carcinomas. High expression of this gene set was also observed in primary tumors of patients with reduced bone or lung metastasis-free survival, suggesting that the common program of v-Src transformed cells is enriched for genes associated with a more aggressive tumor phenotype. The definition of this v-Src gene signature provides a set of biomarkers for the identification of tumors with an aggressive phenotype and the framework for the study of cellular properties conferred by high Src kinase activity in human tumors.

## Methods

### Cell culture and RSV strains

Chicken embryo fibroblasts (CEF) were isolated from day 10 embryos while chicken neuroretinal cells (CNR) were isolated from the retina of 7 day old embryos, as described before [27]. CNR cells do not proliferate in the absence of serum or a transforming v-Src kinase and therefore were infected with the *wt *Schmidt Ruppin-A strain (SR-A) of RSV or with the temperature-sensitive mutant *ts *NY72-4 RSV. CEF were infected with the RSV strains *wt *SR-A, *ts *NY72-4 and *td *NY315 or with RCASBP-A, a derivative of avian sarcoma viruses lacking a viral Src gene. All viruses belong to the A sub-group of ASV. Characterization of the *ts *NY72-4 and *td *NY315 RSV mutants were described before [28,29]. CNR and CEF were cultured in high glucose Richter's modified MEM medium (HyClone #SH30601) supplemented with 5% cosmic calf serum (HyClone #SH30087.03), 5% tryptose phosphate broth, glutamine, penicillin and streptomycin. All studies were performed with actively dividing cells cultured in medium replenished the day before sample preparation to avoid starvation and acidosis of the transformed cells. Populations of *ts *NY72-4 RSV-infected CNR cells were expanded at the permissive temperature of 37.5°C while CEF were cultured at the non-permissive temperature of 41.5°C until transferred to the permissive temperature of 37.5°C to induce transformation. For PI3K inhibition, CEF were treated for 8 hours with either a 100 nm dose of wortmannin (Biomol Research Laboratories, Pennsylvania) or a 15 μM dose of LY290042 (Biomol Research Laboratories, Pennsylvania). Animal use was approved by the McMaster University Animal Research Ethics Board (AUP#05-06-26) and conducted in accordance with the guidelines of the Canadian Council on Animal Care.

### RNA isolation and northern blotting analysis

Total cell RNA was isolated using Trizol reagent (Invitrogen). Ten μg of total RNA was loaded per well and separated on a 1.2% agarose gel containing 3.75% formaldehyde. RNA was transferred to nylon membrane (Schleicher & Schuell) by capillary transfer, cross-linked, and probed with randomly primed radiolabeled DNA fragments as described before [30]. Signals detected on northern blots were quantified directly by phosphor-imaging using a Storm 820 phosphorimager (Molecular Dynamics) or by scanning autoradiographs on a Umax Astra 1220 U scanner. Images were quantified using ImageJ [31] and adjusted for loading using GAPDH.

### Gene profiling analyses

RNA samples were isolated using Trizol (Invitrogen). RNA quality was assessed by gel electrophoresis and examined with a Bioanalyzer (Agilent). All RNA samples were first analyzed by northern blotting analysis and probed for IL8 and GAPDH expression. RNA samples with a RNA Integrity of less than 9.7 were discarded. Microarray experiments were conducted at the Toronto Centre for Applied Genomics (TCAG) at the Hospital for Sick Children (Toronto, Canada). Biotinylated cRNAs were generated at TCAG and hybridized to Affymetrix Chicken GeneChip arrays using standard Affymetrix protocols (EukGE-WS2v4). GeneChips were scanned using the Affymetrix GeneChip Scanner 3000. Feature intensity was quantified using Command Console software and exported to CEL format.

CEL files were analysed using dChip software version 2007 [32]. Array data were normalized against the median intensity array for each experiment using the invariant set normalization method [33]. Median array intensities for the three experiments were scaled to the same value prior to normalization in order to provide comparable probe intensities for inter-experimental comparison. Expression levels were determined by the model-based expression index method using perfect-match probes only [33,34]. Two-fold changes in gene expression between experimental conditions within each experiment were determined using log-transformed expression values and statistical significance of expression was determined using one-way ANOVA (RCASBP/NY315/SR-A comparison using RCASBP(A) and NY315 as control groups) or an unpaired two-tailed t-test (NY72-4 CEF and CNR comparisons). Probe-sets whose p-values were greater than 0.05 (pairwise comparisons) or 0.01 (ANOVA) were excluded from further analysis. False discovery rates in all comparisons were estimated to be less than 3% by permutation (10,000 permutations;[35]). For clustering, redundant genes were removed by identifying probe-sets whose Entrez Gene and/or reference sequence IDs were identical. Probe-sets with the lowest p-values were retained. Gene clustering was performed using unsupervised hierarchal clustering by average Euclidean distance [36]. Venn diagrams were drawn using Vennmaster v. 0.35 [37].

Experimental groups were divided into the following: CEF infected by RCASBP(A), NY315 RSV, or SR-A RSV (experimental group 1); CEF infected by ts NY72-4 RSV and grown either at the permissive or non-permissive temperature (experimental group 2); and CNR cells infected by ts NY72-4 RSV and grown either at the permissive or non-permissive temperature (experimental group 3). A minimum of three biological replicates per experimental condition was used. An additional three RCASBP(A)-infected CEF samples cultured at 37.5°C were used to control for temperature effects (normalized within experimental group 1). Temperature-regulated genes (determined using the same criteria as for the experimental groups) were cross-referenced to all differentially expressed genes from the CEF ts NY72-4 and CNR tsNY72-4 experiments. Genes whose change in magnitude expression was greater than or equal to the same genes found to be differentially expressed in the SR-A/RCASBP(A) experiment were considered to be temperature-regulated and not attributable to v-Src-dependent transformation. This includes 13 genes from the CEF ts NY72-4 and 16 genes from the CNR ts NY72-4 experiments respectively (Additional File 1). Arrays in each experimental group were normalized within said experimental group to prevent skewing of data points from tissue-effects as determined by M-A plots for individual arrays. Array data are accessible through the Gene Expression Omnibus http://www.ncbi.nlm.nih.gov/geo/ using the accession number GSE14489.

### Gene ontology and pathway analyses

Gene ontology (GO) analyses were conducted using the DAVID 2.0 bioinformatics resources [38,39] functional annotation tool using an EASE score of 0.05 and a minimum of two genes per term. Pathway analyses were carried out using Pathway Express software available in the Onto-tools package [40]. Probe-sets associated with significantly differentially expressed genes from each experimental group were converted to the orthologous probe sets from the Affymetrix human U133 Plus 2.0 array. Non-redundant orthologous probe-sets and associated fold change differences (linear-scale) were uploaded to Pathway Express for use in pathway analysis. Pathway analysis was conducted using default settings and only pathways whose corrected gamma-p-value was less than or equal to 0.05 were kept.

### Tumor data analyses

Tumor expression data sets were obtained from the Gene Expression Omnibus at the National Center for Biotechnological Information. Breast tumor data from *Pawitan et al*. [41] and *Ivshina et al*. [42] were used as training data sets (Figure 5, panels Ai and Aii respectively). Breast tumor data from *Minn et al*. [43], colon tumor data from the International Genomics Consortium Expression Project for Oncology http://www.intgen.org/, and breast, lung and ovary tumor data from *Bild et al*. [44] were used as test sets. All clinical data were obtained from the original publications. Orthologous probe-sets (Hu95av2, U133, U133 Plus 2.0 Affymetrix arrays) for the CSR gene set were obtained from NetAffyx (Affymetrix).

To ascertain if up-regulated genes in the CSR set could be used as markers of poor prognosis in human cancer, orthologous probe-sets corresponding to the up-regulated genes in the CSR set were obtained for the Affymetrix platforms used in the training data sets. Only the up-regulated orthologous CSR probe-sets were used for clustering of the Pawitan and Ivshina training sets. Unsupervised gene and sample clustering (Pearson correlation distance metric [36]) was performed on the two training data in order to identify clusters of tumors with similar gene expression profiles. Each tumor cluster was subjected to survival analysis in order to identify tumor clusters associated with poor survival. Since the objective was to identify up-regulated CSR genes associated with poor prognosis, the genes whose expression was statistically significantly higher in the poor-prognosis tumor set versus the other three sets were chosen as candidates for the aggressive gene signature (t-test, single-tailed, p < 0.05). The common set of 42 genes identified from the two training sets defined the aggressive gene signature.

For the test data, the sets of tumors expressing high levels of the aggressive signature were defined as the upper quartile of tumors from any given test set with the highest average gene-wise mean-centered expression of the aggressive signature set. Survival analyses were carried out using the Kaplan-Meier product limit estimator method [45] and statistical significance was evaluated using the log-rank test.

### Immunofluorescence and western blotting

CEFs were grown to sub-confluence on glass slides, rinsed with PBS twice and fixed in 1% formaldehyde/PBS for ten minutes. Cells were washed three times for ten minutes in PBS and incubated with anti-CD44-FITC antibody (Abcam, cat. # ab24907) for 2 hours at room temperature and washed three times for ten minutes in PBS. Cells were visualized on a Nikon Eclipse TE2000-U inverted microscope. Western blotting was performed as described before [46] using the following antibodies: anti-GSK3-β and phospho-S9-GSK3-β (Cell Signaling #9332 and 9336S), anti-heme oxygenase 1 (Stressgen, #SPA-896), anti-PKB (New England Biolabs #9272), anti-phospho-PKB (Cell Signaling #9271), anti-Erk1 (Santa Cruz Biotechnology #sc-94), and anti-Twist1 (Santa Cruz Biotechnology #sc-15393). Autoradiographs were scanned on a Umax Astra 1220 U scanner, quantified using ImageJ and corrected for loading using Erk1 [31].

### Preparation and cloning of DNA fragments

RNA was isolated from 10^7 ^CEFs using Trizol reagent (Invitrogen) as per manufacturer's directions. One μg of RNA was reverse transcribed (Invitrogen SuperScript RT) and treated with RNAse A (Invitrogen). Double-stranded DNA probes for northern blotting analyses were PCR amplified using Promega GoTaq and the appropriate forward/reverse primers (Table 1). DNA fragments were gel-purified and cloned into pCR2.1-TA (Invitrogen) vector as per manufacturer's directions. All cloned products were verified by sequencing at the MOBIX sequencing facility, McMaster University. CEF4/IL8 and glyceraldehyde-3-phosphate dehydrogenase (GAPDH) probes were generated as described previously [30].

**Table 1:** Primer sequences used for the amplification of probe DNA.

Gene	Forward primer	Reverse primer
CD44	TTACTCCGTACTCACATATGCC	CGTCACATGCTCCTGTTCG
DKK3	AAAACCCAGCATACACACTGC	CAGACTTCACACCTGCTTGG
HMOX1	CTGCCCTGGAGAAAGACTTG	AAGCTCTGCCTTTGGCTGTA
ITGA1	CTCTTCTCTACATTACGACG	ATTTTCTTCTTCAGTGGC
ITGA6	GTAATGGCAAATGGCTG	GAACGCTGGAAGAACC
ITGA8	TGGAAAGAGGGAAGAGC	AAGAAGATTGGTGGAAGG
NOV	ATGAAGTGCTCCTGGGAGG	GACATGGGATCTAATGGCTGG
PLCPI	CTCCTCAGAACCACTGCACA	TTCAAGTGTATTTTATTCTCCTGCAT
THBS2	GGGTTATTCGCCACCAAGG	TAGACCTAATCGTCCACCAGC
UPP1	TATGAAGGACAGGGCAGGTT	TTTCAAACGTCACAGCAAGC
VIP	TAGAAAACGAGTTAGCTCCCAGGA	AGAGTTTGCTAGGTGTCCTTCAGA

## Results

### Identification of Transformation-Regulated genes in v-Src transformed CEF

To identify genes regulated in a transformation-dependent manner, we characterized the gene expression profiles of CEF transformed by the wt RSV strain Schmidt Ruppin - group A (SR-A) or infected with the transformation-deficient viruses NY 315 RSV or RCASBP(A). NY315 RSV is a group A virus encoding a catalytically active deletion mutant of the v-Src kinase lacking amino acids 2-14 of the SH4 domain. As a result of this deletion, the v-Src kinase of NY315 RSV is not myristoylated, does not associate with the plasma membrane and is non-transforming [29]. RCASBP(A) is a group A replication competent virus of the avian sarcoma virus family lacking an oncogene [47]. Total cellular RNA was isolated and analyzed on the Affymetrix chicken GeneChip, an array comprising 37,703 probe sets representing 19,881 UniGene clusters. A total of 3254 probe sets, corresponding to 2904 unique genes were differentially expressed by two fold or greater in cells infected by either NY315 or SR-A RSV (Additional File 2). Within regulated sequences, 2455, 1691 and 730 transcripts were differentially expressed in pair-wise comparisons between SR-A and RCASBP(A), NY315 and RCASBP(A), and SR-A and NY315 infected CEF, respectively.

**Figure 1:**
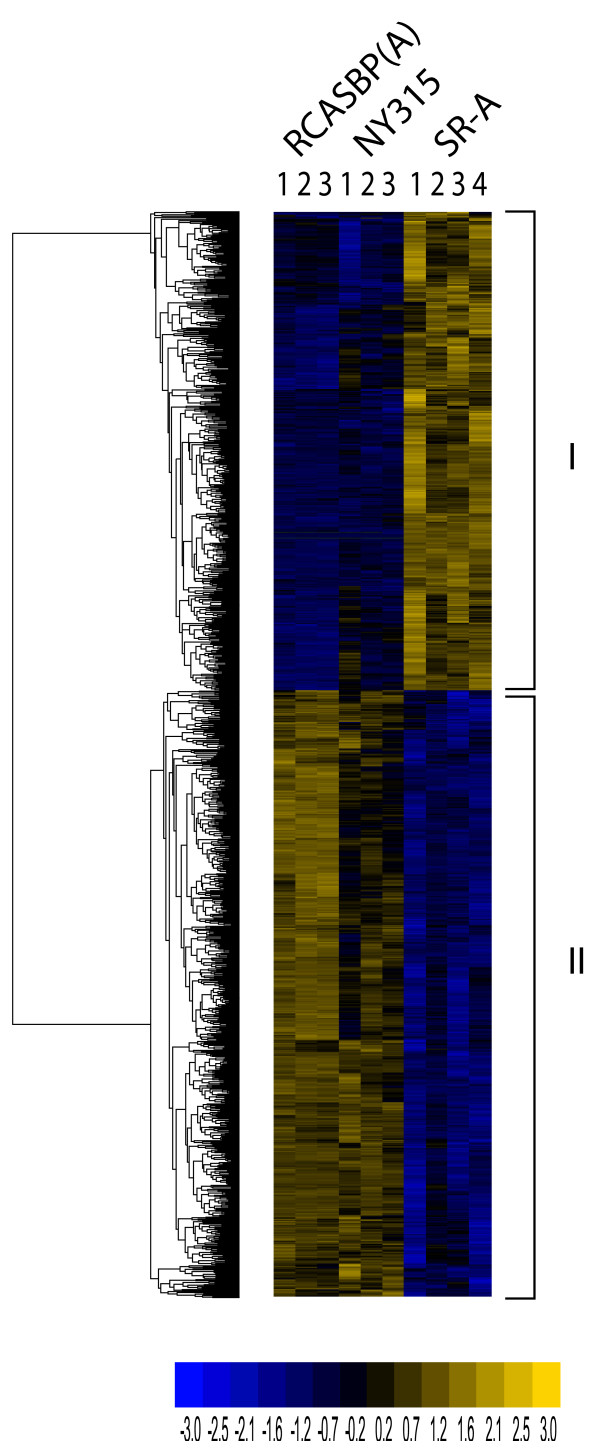
**Transformation regulated (TR) genes belong to two clusters of differentially expressed genes in SR-A RSV transformed CEF**. Unsupervised hierarchal clustering was performed on the Transformation-Regulated (TR) gene set revealing transformation responsive genes clustering into one of two approximately equal sized clusters. Class I includes genes up-regulated during transformation representing approximately 44% of the TR genes while class II comprises the down-regulated genes (56% TR genes). The color scale indicates standard deviations from mean centered gene expression values.

Genes regulated in a transformation-dependent manner were identified by one-way ANOVA (p≤0.01) using RCASBP(A) and NY315 as control groups and defined by probe sets with differences in expression greater or equal to two-fold between any two conditions. Unsupervised hierarchal clustering shows that transformation responsive genes cluster to one of two groups, either up-regulated or down-regulated in SR-A RSV transformed CEF but not in the other two conditions (Figure 1). A total of 1095 transcripts corresponding to 418 up-regulated and 535 down-regulated genes were identified in this analysis, defining a set of genes regulated in a transformation-dependent fashion. Approximately 15% of these genes were differentially expressed by five fold or more. We refer to this set of genes as the **T**ransformation-**R**egulated genes (TR genes) of v-Src transformed CEF (Additional File 3).

**Figure 2:**
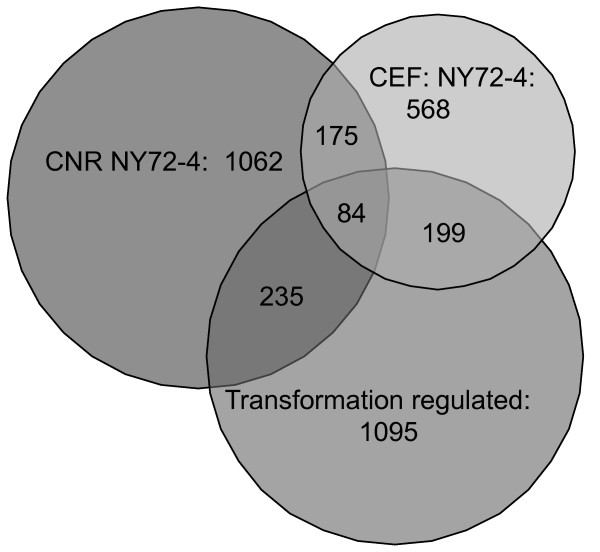
**Euler representation of genes differentially expressed between control and v-Src transformed cells**. Comparison of gene sets differentially expressed by two-fold between CEF or CNR cells infected with the temperature sensitive mutant NY72-4 RSV, or the set of Transformation-Regulated (TR) genes defined in Additional File 3. Numbers indicate total probe sets.

Differentially expressed genes were also characterized in CEF infected with the temperature sensitive (*ts*) mutant NY72-4 RSV. A pair-wise comparison of CEF infected with NY72-4 revealed that 568 transcripts encoded by 477 unique genes (261 up- and 216 down-regulated) were differentially expressed at the permissive versus non-permissive temperatures (Figure 2 and Additional File 4). Cross-referencing of the gene lists provided by the SR-A/NY315/RCASBP(A) and NY72-4 RSV analyses revealed a set of 145 non-redundant genes (or 199 transcripts) corresponding to 84 down- and 61 up-regulated genes associated with cell transformation (Additional File 5).

To control for the effect of temperature change (cold shock), we compared the profile of RCASBP(A) infected CEF at the permissive temperature of 37.5°C and non-permissive temperature of 41.5°C (Additional File 1). Few genes were differentially expressed by a factor of three or more at the two different temperatures. However, some of the highly v-Src responsive genes (HMOX1, UPP1, Aquaporin 1, for instance) were also regulated by temperature change. Genes affected by temperature and *wt *v-Src, as determined by the analysis of control and SR-A RSV-transformed CEF at 41.5°C, indicate a sub-class of genes that are both stress-responsive and v-Src-regulated.

### Identification of v-Src regulated genes in chicken neuroretinal (CNR) cells

In vertebrates, the c-Src kinase is widely expressed and detected in most tissues. In addition, v-Src is capable of transforming a variety of cell types *in vitro *[48-54]. To compare the biological response of different cell types transformed by v-Src, we characterized the patterns of gene expression of chicken neuroretinal (CNR) cells infected with the temperature-sensitive mutant *ts *NY72-4 RSV. Unlike CEF, neuroretinal cells do not proliferate *in vitro *unless transformed by an oncoprotein such as v-Src and cultured in serum-containing medium [55]. Therefore, *ts *NY72-4 RSV infected CNR are quiescent at the non-permissive temperature of 41.5°C but are actively dividing and transformed at the permissive temperature of 37.5°C. Gene profiling analyses of CNR cells identified 1062 transcripts, consisting of 485 up- and 577 down-regulated RNA species, with a two-fold or greater difference in gene expression between the two temperatures (Additional File 6). A significant fraction of the v-Src regulated genes identified in *ts *NY72-4 RSV (31%) and SR-A/NY315/RCASBP(A) infected CEF (21%) overlapped with the corresponding set of genes in CNR cells (Figure 2). The total number of transcripts (probe sets) and genes differentially expressed in the three systems of v-Src transformation is provided in Table 2. Together, the total genes regulated in these three sets represent approximately 6% of the protein-coding genes in the chicken genome. When all three systems are compared, a group of 84 common genes are identified as v-Src regulated (Figure 2; Additional File 7).

**Table 2:** Summary of probe sets found differentially regulated in all three systems of v-Src regulation.

	Total probe-sets			Unique transcripts		
	
Analysis	up	down	total	up	down	Total
Transformation regulated	483	612	1095	418	535	953
CEF NY72-4	319	249	568	261	216	477
CNR NY72-4	485	577	1062	444	503	947

Number of transcripts differentially expressed in the Transformation-Regulated dataset (RCASBP(A), NY315, SR-A RSV analysis) and the ts NY72-4 RSV infected CEF and CNR cells is shown. Numbers under the *total probe-sets *heading indicate the total number of Affymetrix probe-sets hybridizing to differentially expressed transcripts, whereas *unique transcripts *do not include redundant probe-sets whose Entrez Gene and/or reference sequence ID were identical.

A second group of 91 genes was regulated in ts NY72-4 RSV infected CEF and CNR cells but not in SR-A RSV transformed CEF (Additional File 8). Up-regulated genes in this list encode several important regulators of cell proliferation or behavior including ornithine decarboxylase (ODC), osteopontin, hyaluran synthase 2 (HAS2), cyclin A and cyclin E2. In contrast, the cyclin kinase inhibitor p27^Kip1 ^was down-regulated in NY72-4 RSV transformed CEF and CNR cells. Since CNR cells are amplified and cultured at the permissive temperature for extensive periods of time, it is unlikely that the regulation of these genes represents a transient effect of ts v-Src activation. A more likely explanation for this discrepancy is that differences in the structure of the v-Src kinase encoded by NY72-4 and SR-A RSV account for this specificity in gene regulation. It is also likely that the list of genes defined above (Additional File 7) is an under-representation of the gene cohort controlled by v-Src in CEF and CNR cells. A more accurate description of this class of genes can be obtained by combining the genes described in Additional File 7 and the genes regulated uniquely in NY72-4 RSV transformed CEF and CNR cells (Additional File 8). This combined list would then consist of a total of 175 genes that we define as the **C**ommon set of v-**S**rc **R**egulated genes or **CSR **genes.

### Validation and characterization of v-Src regulated genes

**Figure 3:**
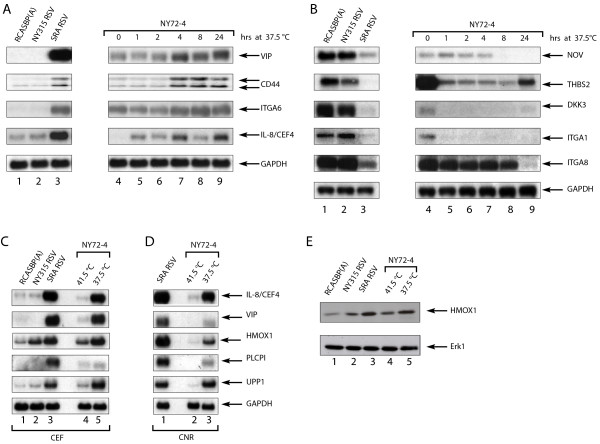
**Validation of gene profiling results by northern and western blotting analyses**. Steady state transcript levels for a selected set of v-Src regulated genes were determined by northern blotting analyses (A-D). CEF infected with RCASBP(A), NY315 or SR-A RSV were maintained at 41.5°C for the duration of the analysis while NY72-4 RSV infected cells were cultured at the permissive and non-permissive temperatures of 37.5°C and 41.5°C, respectively, for the indicated period of time. AB) CEF infected with NY 72-4 RSV were grown at the non-permissive temperature of 41.5°C and transferred to 37.5°C for the indicated period. C-D) CNR cells infected with NY 72-4 RSV were grown at the permissive temperature of 37.5°C and either maintained at this temperature or transferred to 41.5°C for a 24 hr period before RNA isolation. CNR cells transformed by SR-A RSV were kept at 41.5°C. RNA loading was assessed by probing for GAPDH. E) Western blotting analysis of heme oxygenase 1 (HMOX1) in CEF. Protein samples were prepared from CEF infected RCASBP(A), NY315 or SR-A RSV at 41.5°C. CEF infected with NY 72-4 RSV were either kept at the non-permissive temperature of 41.5°C or transferred to 37.5°C for 24 hrs before lysis. Erk1 was used as a control to assess protein loading.

The expression of v-Src regulated genes was confirmed by northern blotting analyses. In this study, the expression of genes identified by gene profiling was compared to that of previously characterized markers of Src transformation including IL8/CEF-4, CD44 and Nov [10,14,56-58]. For all up and down-regulated genes selected, little difference was observed between non-transformed CEF infected with the replication competent virus RCASBP(A) and CEF infected with the transformation-deficient virus NY315 RSV (Figure 3A). As reported before, the kinetics of IL8/CEF-4 RNA accumulation was biphasic and characterized by the rapid accumulation of IL8/CEF-4 (within 1 hr) followed by a temporary reduction of RNA level between 4 and 8 hrs of temperature shift [59]. Other RNA species (VIP, CD44, integrin-α-6) accumulated more slowly i.e. between 2 and 4 hrs following *ts *v-Src activation and did not show the transient down-regulation observed for the IL8/CEF-4 mRNA. IL8/CEF-4 and CD44 were both induced in cells treated with cycloheximide indicating that the different kinetics of RNA accumulation do not necessarily reflect a different requirement for *de novo *protein synthesis (data not shown).

Cell heterogeneity was assessed by looking at the surface expression of CD44 by immunofluorescence (Additional File 9). A strong CD44 signal was observed in SR-A RSV transformed CEF but was absent in cells infected with RCASBP(A) and NY315 RSV. All cells expressing the ts mutant of v-Src (NY72-4 RSV) were also positive for CD44 expression at the permissive temperature. Low but detectable signals for CD44 expression were also observed in ts NY72-4 RSV-infected cells at the non-permissive temperature. This may be indicative of a certain degree of leakiness of the ts v-Src kinase, an observation accounting for the lower number of v-Src regulated genes identified in this system.

### Characterization of the common set of v-Src regulated genes in CEF and CNR cells

Differences in the profiles of v-Src regulated genes in CEF and CNR cells reflect in part the proliferative and differentiation state of these cells since CNR cells do not proliferate when non-transformed and are partially committed to differentiation [55,60]. However, a common program of gene expression was also evident in the profiles of v-Src transformed CEF and CNR cells. Genes such as IL8/CEF4, VIP, HMOX1, PLCPI and UPP1, were all activated in v-Src transformed CEF and CNR cells. HMOX1 also provided an example of a gene with partial activation in CEF infected with NY315 RSV, both at the RNA and protein level (Figure 3C &3E).

A comparative analysis of v-Src regulated mRNA species examined by northern blotting and microarray profiling was performed for all mRNA species included in Figure 3. This study revealed a strong correlation (Spearman ρ of 0.83, p < 0.0001) between northern and microarray gene expression estimates. A regression coefficient of 0.91 indicates a nearly 1:1 correspondence between northern blotting and microarray gene expression values (Additional File 10, Additional File 11). Collectively the results of the immunofluorescence, northern, and western blotting analyses validated the expression of a sub-set of genes identified in the gene profiling studies and illustrated the existence of a common program of gene expression controlled by v-Src in different cell types.

### Signaling pathways and biological processes regulated during v-Src transformation

Pathways potentially regulated by v-Src-induced changes in gene expression were identified by Pathway Express software [40]. Pathway Express estimates whether genes differentially expressed in a given data set impact a given pathway by incorporating fold-change of gene expression, where those genes are in the hierarchy of a pathway and whether those genes are found to be statistically overrepresented in that pathway. Using this methodology, "ECM-receptor interaction", "focal adhesion" and "phosphatidylinositol signaling" were identified as pathways most significantly altered in all three data sets (corrected γ p-value ≤ 0.05; Table 3). TGFβ signaling was also identified in CEF but not CNR cells. Several pathways were also identified in a single data set and, in particular, in the large cohort of the Transformation-Regulated gene set. Thus, "leukocyte transendothelial migration", "complement and coagulation cascades", "cell adhesion molecules", "MAPK" and "Toll-like receptor signaling" pathways were all identified in this data set (Additional File 12).

**Table 3:** Pathway Express output summary.

		#Input genes in pathway			Corrected γ p-value		
		
Pathway Name	# genes in pathway	TR	CEF NY72-4	CNR NY72-4	TR	CEF NY72-4	CNR NY72-4
ECM-receptor interaction	87	19	10	14	1.83E-11	3.37E-06	3.14E-07
Focal adhesion	195	26	14	19	6.87E-11	1.44E-06	3.47E-06
Phosphatidylinositol signaling system	77	3	1	3	7.71E-10	4.80E-11	1.71E-03
TGF-beta signaling pathway	84	10	7	N.D.	2.90E-06	2.12E-04	N.S.
Regulation of actin cytoskeleton	208	19	N.D.	11	1.27E-05	N.S.	5.03E-03
Small cell lung cancer	86	12	6	8	2.56E-05	9.19E-03	1.26E-02
Complement and coagulation cascades	69	4	2	N.D.	1.31E-03	2.15E-02	N.S.
Epithelial cell signaling in Helicobacter pylori infection	67	3	1	N.D.	2.80E-03	1.09E-05	N.S.
Leukocyte transendothelial migration	116	7	2	7	3.19E-03	1.73E-02	2.75E-02
Type II diabetes mellitus	44	4	1	N.D.	2.60E-02	2.85E-02	N.S.

Common pathways found to be dysregulated in the Transformation-Regulated (TR), CEF NY72-4 and CNR NY72-4 gene sets are shown. The *number of genes in the pathway *refers to the number of genes in the associated KEGG pathway (Kyoto Encyclopedia of Genes and Genomes, http://www.genome.jp/kegg/; [102]). *Input genes *refer to the number of differentially expressed genes that were found in that pathway. Corrected γ p-value is a measure of significance as calculated by Pathway Express. N.D. and N.S. indicate *not determined *and *not significant *(corrected γ p-value > 0.05) respectively.

Pathway Express stresses the regulatory nature of a gene product. This is best illustrated by the genes encoding the pro-survival regulatory and catalytic sub-units of PI3K, which are part of the "focal adhesion", "PI3K" and "leukocyte transendothelial migration" pathways and are hallmarks of several cancer types (ex. "small cell lung cancer"). The up-regulation of both the catalytic and regulatory subunits of PI3K in SR-A RSV transformed CEF implies that the pathway is strongly activated in these cells (Additional File 3). This was confirmed by looking at the phosphorylation status of PKB/Akt and GSK3β, two downstream targets of the PI3K pathway. High levels of phospho-PKB and phospho-GSK3β were detected in SR-A RSV transformed CEF but not in cells infected with RCASBP(A) or NY315 RSV, even when these cells were actively cycling (Additional File 13). The signal was reduced in cells treated with the specific inhibitor LY294002, indicating that phosphorylation of these proteins was dependent on the PI3K pathway.

**Figure 4:**
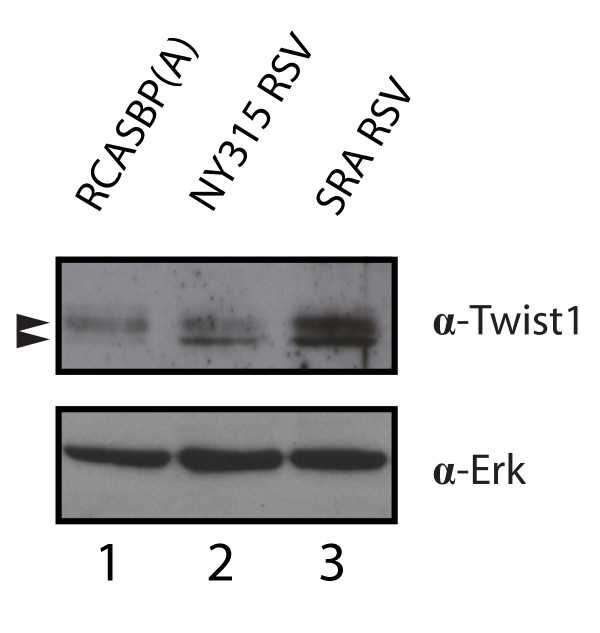
**Up-regulation of Twist1 in v-Src-transformed CEF**. Twist1 protein levels are up-regulated in v-Src transformed CEF. Two Twist1 immunoreactive protein species of 26 kDa of molecular weight are detected in normal and v-Src transformed CEF.

Other important regulatory factors are included in the list of Transformation-Regulated genes. An example is provided by Twist1, a bHLH transcription factor promoting metastasis and capable of inducing the epithelial-to-mesenchymal transition (Additional Files 3 &4;[61]). The up-regulation of Twist1, observed as a 26 kDa doublet by western blotting, was confirmed in SR-A RSV transformed CEF (Figure 4).

### The Common Set of v-Src Regulated (CSR) genes includes a subset of genes associated with reduced disease-free survival in human cancer

To test whether v-Src-induced changes in gene expression correlate with reduced disease- or metastasis-free survival in human cancers, we undertook a clustering-based enrichment regime to identify up-regulated genes of the CSR set associated with this phenotype. To this end, two independent breast carcinoma datasets with clinical information were selected and used as training sets. Mammary tumor sets were used because of the abundance of good quality array data obtained on more recent Affymetrix platforms and because a large proportion of breast carcinomas (>70%) exhibit high Src activity [3]. It was also reasoned that CSR genes were more likely to be regulated in tumors because their expression is controlled by v-Src in different cell types (i.e. CEF and CNR cells). The aggressive gene signature, defined by clustering analysis, was then tested against additional, independent tumor datasets to assess if it could be used to predict reduced disease-free survival.

**Figure 5:**
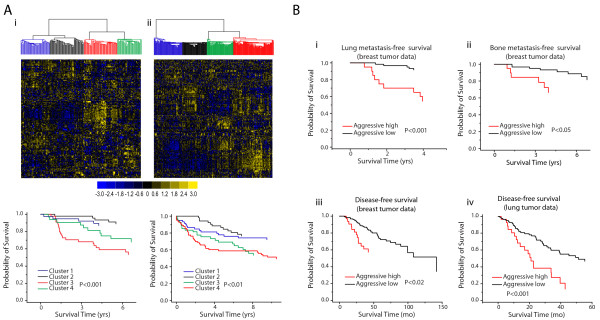
**A subset of CSR genes predicts poor prognosis in human tumors**. A) Hierarchal clustering of two breast tumor datasets with respect to up-regulated CSR genes reveals tumor sample clusters associated with low disease-free survival (cluster 3 in panel i and cluster 4 in panel ii). The color scale beneath the heat maps indicates standard deviations from mean centered gene expression values. Colors of survival curves correspond to the colored clusters indicated in the heat maps above each survival plot. B) A common set of up-regulated genes associated with low-survival clusters in panel A, termed the aggressive tumor gene signature (Table 4), correlates with poor prognosis in several patient cohorts. Tumor clusters expressing high levels of the aggressive signature genes (aggressive high) correlate with lower lung and bone metastasis-free survival in patients with breast tumors (i and ii), as well as with lower disease-free survival in separate cohorts of patients with breast (iii) or lung tumors (iv).

Probe-sets corresponding to orthologous genes from the up-regulated CSR set and tumor samples from the two independent breast carcinoma datasets [41,42] were clustered to identify samples containing unique up-regulated gene clusters associated with poor disease-free survival. Survival analyses of these tumor datasets revealed tumor sample clusters associated with higher disease relapse (Figure 5A). Figure 5Ai shows that sample cluster 3 has poorest estimated mean disease-free survival (5.6 years) compared to the other three clusters (7.6, 7.8, and 6.8 years; clusters 1, 2 and 4 respectively; P < 0.001). In Figure 5A, panel ii, cluster 4 has the poorest estimated mean survival compared to clusters 1, 2 and 3 (7.8, 10.1, 10.7 and 8.4 years respectively; P < 0.01). CSR genes that were up-regulated in the low-survival cluster in relation to the other tumor clusters of each tumor data set were cross-referenced to each other to generate an aggressive tumor gene signature (Table 4, see methods). This set contains 42 unique genes overlapping with an 80-87% concordance with respect to the genes found differentially up-regulated in the two tumor data training sets. This striking overlap represents over 50% of the 80 up-regulated CSR genes (Additional Files 7 &8) indicating that the common program of v-Src gene expression is enriched for genes associated with an aggressive tumor phenotype. Functional classification of these genes showed greatest enrichment for gene ontology biological processes (GOBP) such as "Cell Cycle" ("M-phase", "Mitosis", "Cell Division"), "DNA Metabolic Process" ("Response to DNA damage stimulus"), "Response to Stress", "Cell Proliferation" and "DNA Replication". (Additional File 14)

**Table 4:** v-Src aggressive tumor gene signature.

Gene symbol	Gene name
ATAD3A	ATPase family, AAA domain containing 3A
C13orf3	chromosome 13 open reading frame 3
CCNA2	cyclin A2
CCNE2	cyclin E2
CEP55	centrosomal protein 55 kDa
CSTA	cystatin A (stefin A)
E2F8	E2F transcription factor 8
EAF2	ELL associated factor 2
EXO1	exonuclease 1
GAR1	GAR1 ribonucleoprotein homolog (yeast)
HELLS	helicase, lymphoid-specific
HMOX1	heme oxygenase (decycling) 1
HSP90AB1	heat shock protein 90 kDa alpha (cytosolic), class B member 1
IL8	Interleukin 8
ITGA4	integrin, alpha 4 (antigen CD49D, alpha 4 subunit of VLA-4 receptor)
KIF11	kinesin family member 11
KIF2A	kinesin heavy chain member 2A
LBR	lamin B receptor
MPP1	Membrane protein, palmitoylated 1, 55 kDa
NASP	nuclear autoantigenic sperm protein (histone-binding)
NOC2L	nucleolar complex associated 2 homolog (S. cerevisiae)
NOP14	NOP14 nucleolar protein homolog (yeast)
NPM3	nucleophosmin/nucleoplasmin, 3
ODC1	ornithine decarboxylase 1
PDCD6	programmed cell death 6
PLAU	plasminogen activator, urokinase
RIOK3	RIO kinase 3 (yeast)
RRM1	ribonucleotide reductase M1
RRM2	ribonucleotide reductase M2 polypeptide
SHC4	SHC (Src homology 2 domain containing) family, member 4
SLC2A14	solute carrier family 2 (facilitated glucose transporter), member 14
SLC2A3	solute carrier family 2 (facilitated glucose transporter), member 3
SLC36A4	solute carrier family 36 (proton/amino acid symporter), member 4
SOCS1	Suppressor of cytokine signaling 1
TRIP13	thyroid hormone receptor interactor 13
TTC35	tetratricopeptide repeat domain 35
UHRF1	ubiquitin-like with PHD and ring finger domains 1
UPP1	uridine phosphorylase 1
USP1	ubiquitin specific peptidase 1
USP18	ubiquitin specific peptidase 18
VRK1	vaccinia related kinase 1
ZDHHC21	zinc finger, DHHC-type containing 21

Aggressive gene signature used as a predictor of poor prognosis as determined by hierarchal clustering enrichment regime (see text, Figure 5).

To assess whether the aggressive tumor gene signature could be used to predict poor disease-free survival, six independent tumor datasets were interrogated to determine if high expression of the 42 genes defining this signature was associated with a poor outcome. By comparing the top 25% of tumors expressing the highest levels of the aggressive gene signature against the bottom 75%, four data sets out of six showed that patients with tumors characterized by high expression of the 42 gene cohort have reduced disease-free or metastasis-free survival (Figure 5B). Reduced relapse-free survival was seen in the breast and lung carcinoma datasets while reduced lung and bone metastasis-free survival was observed in two other breast tumor datasets.

Analysis of tumors expressing the highest levels of the aggressive signature genes in the breast tumor set from *Minn et al*., [43] indicated that the patients from whom the tumors were derived had reduced disease-free survival with respect to lung metastasis (mean estimated survival 5.4 years versus 10.1 years, P < 0.001; Figure 5Bi). Similarly, using bone metastasis data from the same study, the high aggressive signature expressing tumors were associated with reduced metastasis-free survival in patients (mean estimated survival 6.4 years versus 9.3 years, P < 0.05; Figure 5Bii). Analysis of a second breast tumor data set [44] also shows that patients whose tumors express high levels of the aggressive signature genes have a reduced disease-free survival (mean estimated survival 71 months versus 106 months, P < 0.02; Figure 5Biii). Lung tumor data from the same study indicates a similar trend where patients with tumors expressing high levels of the aggressive signature have reduced disease-free survival (mean estimated survival 29 months versus 54 months, P < 0.001, Figure 5Biv). Analysis of ovarian tumor data from the same study however did not yield any statistically significant difference (data not shown). Since survival data was not available for the colon carcinoma dataset, we asked whether high expression of the aggressive tumor gene signature could identify patients with distal metastases. Analysis of the relative proportions of metastasis in patients exhibiting high expression of the aggressive signature to those that did not, failed to show any statistically significant difference (data not shown). Taken together, these data show that a subset of the v-Src-regulated CSR genes correlates with poor prognosis in patients with breast tumors, exhibiting some specificity for metastasis to lung and bone.

## Discussion

### Definition of a gene signature for v-Src transformation of primary cells

Previous gene profiling studies of v-Src transformed cells have relied principally on the use of immortalized rodent cell lines that may not reflect the full spectrum of biological processes altered during v-Src transformation [24,25]. In this study, we used transformation-deficient (NY315 RSV) and temperature sensitive mutants of the Rous sarcoma virus (NY72-4 RSV) to identify gene products regulated in a transformation-dependent manner in primary cells. Further, all experiments were performed in conditions promoting asynchronous cell division and limiting acidosis. A separate study of RSV transformed CEF was reported previously [23]. However, most of the genes identified in our studies (80%) were not included in the gene set described by these investigators. Moreover, the results of this study differ from ours in some important aspects. First, Masker and co-workers did not assess the transformation-dependence of differentially expressed genes since their analysis was restricted to a pairwise comparison of SR-A RSV transformed CEF and CEF infected with a replication competent virus devoid of any oncogene. Second, several established markers of v-Src transformation such as IL8/CEF-4 and CD44 [10,14,56], were poorly regulated in their experimental conditions and did not pass the significance criteria of the analysis. Third, Masker and co-workers identified some transcription factors such as c-Jun and c-Myc, as being up-regulated by v-Src while we did not. We previously established that early immediate genes such as c-Myc are activated by v-Src in quiescent cells but are not differentially expressed when actively dividing cells are compared [14]. Therefore, some of these differences may reflect the experimental conditions of the studies and, in particular, the conditions of cell culture.

A study performed with ts NY72-4 v-Src identified a limited number of v-Src regulated genes in NIH 3T3 fibroblasts [26]. Key transcriptional regulators identified by these authors, such as Hmga2 (high mobility group AT-hook2) and Id4 (inhibitor of DNA binding 4), are also included in the list of v-Src-regulated genes in CNR cells (Additional File 6). In addition, Liu and co-workers described the regulation of important regulators of the cell cycle and angiogenesis by SSeCKS, a metastasis-suppressor gene down-regulated by v-Src [26]. Interestingly, tumor suppressor genes such as DKK3 and gamma FBPα, the avian homolog of HIC-1 (hypermethylated in cancer 1), were repressed markedly by v-Src in CEF. Thus, the abrupt down-regulation of tumor-suppressor genes may be required for v-Src-dependent cell transformation (Figure 3 and Additional File 3; [26]).

### The common set of v-Src regulated genes (CSR genes) is enriched for genes associated with an aggressive tumor phenotype

Since the c-Src kinase is expressed ubiquitously in most vertebrate tissues, we reasoned that genes regulated by v-Src in multiple cell types would better represent targets of this kinase in tumorigenesis. Using CEF and CNR cells, we thus described a common program of gene expression consisting of 175 genes regulated by v-Src in both cell types. This program, referred to as the **C**ommon set of v-**S**rc **R**egulated genes or **CSR **genes, was then used to interrogate several independent tumor data sets with corresponding clinical information. Beginning with data from two independent cohorts of breast carcinomas as training sets, we performed hierarchal clustering analyses to identify a group of v-Src inducible genes highly expressed in tumors associated with reduced disease-free survival (Table 4). Significantly, the majority (80-87%) of the 42 genes included in this group were identified in both independent tumor data sets. Since the CSR gene cohort consists of a total of 80 up-regulated genes, the 42 genes identified in this analysis suggest that CSR genes are highly enriched for genes associated with a more aggressive tumor phenotype. To confirm this finding, we then interrogated several additional tumor data sets including some used previously by other investigators [43,44]. This analysis confirmed the expression of the v-Src inducible gene set in primary tumors of patients with reduced disease-free or metastasis-free survival in four of six tumor data sets, including breast and lung carcinomas (Figure 5). In contrast, no correlation was observed for ovarian and colon carcinomas suggesting tissue specificity of the v-Src aggressive tumor gene signature. A recent report by Zhang and co-investigators shed some light on the role of Src in bone metastasis of breast cancer cells [9]. Knockdown of c-Src in the highly metastatic human BoM-1833 cell line impaired the proliferation of these cells in the bone micro-environment but had no effect on lymph node metastasis in a mouse model. Moreover, these authors provided evidence that Src enhanced the survival of the metastatic cancer cell line by mediating the activation of PKB/Akt in response to CXCR4 activation [9].

Prognostic gene signatures for breast cancer have been described previously by several groups [62-66]. A comparative analysis of the 70-gene, 76-gene and the Gene expression Grade Index (GGI) signatures revealed that they have similar prognostic ability despite limited overlap in gene identity [64]. Recently, *Yu et al*. showed that a common set of biological processes is enriched among these gene signatures even when defined by different genes. For instance, "Cell Cycle" is enriched in the 70-gene, 76-gene and the 97-gene GGI signatures even though cyclin E2 (CCNE2) is the only gene of this pathway common to all three signatures [63,65-67]. Interestingly, CCNE2, the only gene found in more than two signatures [67] was also identified in our aggressive gene signature along with EXO1 and KIF11, found in two other studies [62,63] (Table 4). Functional classification of our 42-gene aggressive signature shows that three of the 16 enriched GOBP terms ("Cell Cycle", "Mitosis" and "DNA replication") are identical to three of the 36 core GOBP terms described by *Yu et al*. Twelve remaining terms from our aggressive signature map to parent terms of one or more of the 36 core terms while two, "blood vessel morphogenesis" and "cell proliferation", are unique to our gene set (Additional File 14). This similarity in enriched processes shared with other breast cancer signatures reinforces the hypothesis that there is a common aggressive tumor "pathway-signature" [67].

### Biological processes defined by the CSR gene signature - DNA damage and the stress response

Several genes of the DNA damage and stress responses are included in the 42 genes of the aggressive tumor signature. DNA damage resulting from the production of reactive oxygen species (ROS) or DNA replication stress has been observed in cells transformed by several oncogenes [68-72]. However, evidence of DNA damage, as indicated by the levels of γ H2AX, is presently lacking in v-Src transformed CEF (our unpublished results). Therefore, the pathways responsible for the production of ROS or replicative stress may be attenuated in these cells. Src-transformed cells are known to be more resistant to conditions of oxidative stress and the induction of genes such as HMOX1 or UPP1 enhance cell survival in conditions of hypoxia or glucose depletion i.e. in conditions promoting the production of ROS [73-77]. Therefore, genes of the aggressive tumor signature may limit the effects of oncogenic stress and provide a survival advantage to the cell.

Several genes identified in this study were also regulated in response to temperature change (cold shock). Since temperature change had a modest effect on the activation of these genes with induction levels of 3-fold or less (Additional File 1), and since all v-Src regulated genes affected by temperature change were also identified in the RCASBP/NY315/SR-A RSV analysis at 41.5°C, it is unlikely that these genes represent a class of genes regulated uniquely as a result of temperature change. Genes like HMOX1, Aquaporin 1 and the metallothioneins MT2A and 3 are induced in response to a wide variety of stress conditions and are widely regarded as general stress response genes. Since HMOX1, Aquaporin 1 and UPP1 are activated by temperature change and are included in the Common Set of v-Src Regulated (Additional Files 7 &8), they may be part of a general and previously unrecognized stress-response program controlled by v-Src.

### Biological processes affected by v-Src transformation: Motility and Invasiveness

The large cohort of Transformation-Regulated genes (TR genes; Additional File 3) includes several genes that may contribute to a more aggressive tumor phenotype. Src-transformed CEFs are highly motile and characterized by structures mediating cell migration called podosomes [78,79]. Like the related invadopodia described in other cancer cells, the podosomes are actin-rich structures closely associated with adhesion molecules and ECM-degrading enzymes such as the matrix metalloproteinases MMP-1, MMP-2 or MMP-9 [80]. In CEF, v-Src transformation stimulated the expression of four critical regulators of actin polymerization and podosome formation, namely N-WASP, cortactin, gelsolin and the p41 subunit of the actin related protein complex 2/3 (Arp 2/3 subunit 1b-p41; Additional File 3[81-84]). Two of these factors (Arp 2/3 subunit 1b-p41 and cortactin) were also activated in CNR cells indicating that v-Src controls the expression of these genes in diverse cell types (Additional File 6). The gene encoding the p41 subunit of the Arp2/3 complex is frequently amplified in pancreatic cancer and is a regulator of the motility and invasion of pancreatic cell lines [85]. In contrast, caldesmon and transgelin were repressed by v-Src in CEF. Transgelin is a multi-functional protein with roles independent of podosome formation. In particular, transgelin is a negative regulator of MMP-9 expression and a suspected tumor suppressor [86,87]. Independent studies have shown that caldesmon, an actin filament cross-linker, antagonizes the action of the Arp 2/3 complex and is a negative regulator of podosome formation and invasion in transformed cells [88-90]. Therefore, v-Src transformation is characterized by changes in gene expression promoting the dynamic remodeling of the actin cytoskeleton and the assembly of podosomes. It is also significant that wt v-Src induces the expression of the p110δ catalytic subunit, a PI3K isoform required for cell migration in macrophages and breast cancer cells (TR genes; Additional File 3; [91,92]).

### Biological processes affected by v-Src transformation: Epithelial-to-mesenchymal transition and drug resistance

Several studies have linked the expression of v-Src and other Src family kinases to resistance of a variety of chemotherapeutic agents including cisplatin, geftinib, paclitaxel, oxaliplatin and tamoxifen [93-97]. Since chemoresistance is often associated with increased cell motility and invasiveness, it has been suggested that v-Src controls these activities by inducing the epithelial-to-mesenchymal transition (EMT) in tumor cells [8]. In support of this notion, Sham and co-workers reported recently that the up-regulation of the basic-helix-loop-helix factor Twist1 by NF-kB increases chemoresistance of PC3 prostate cancer cells treated with daunorubicin or cisplatin [98]. In a separate report, Cheng *et al*. identified Twist1 and its target Akt2/PKBβ as factors contributing to the metastatic potential of highly invasive breast carcinoma cell lines. In the same study, these investigators showed that Twist1 and Akt2/PKBβ determine the resistance of these cells to paclitaxel [99]. Twist1 is a potent inducer of the EMT and a member of the Transformation-Regulated gene cohort identified in this study (Figure 4 and Additional File 3). Since forced expression of Twist1 is sufficient to induce the EMT in mammary epithelial cells, the regulation of this factor provides a mechanism by which v-Src may induce the EMT and enhance chemoresistance [61]. Twist1 is not the only marker of the EMT identified in this study since N-cadherin mRNA expression was also up-regulated in SR-A transformed CEF (TR genes; Additional File 3).

## Conclusion

Elevated Src kinase activity has been described in several unrelated human tumors and in cells derived from these tumors [2]. In human cancers, high Src activity correlates with progression to a more malignant phenotype and the increased metastatic potential of tumor cells [4,100,101]. In this study, we define a signature of 42 v-Src inducible genes whose expression is associated with reduced disease-free survival in breast and lung cancer patients from several independent studies. In one dataset, this 42 gene signature was also associated with reduced bone and lung metastasis-free survival. Several genes of the larger cohort of Transformation-Regulated Genes (TR genes) have also been associated with features of aggressive tumors such as invasiveness and chemoresistance. These genes provide a set of biomarkers and candidate therapeutic targets for the treatment of patients with tumors characterized by more aggressive behavior. The functional characterization of these genes represents a roadmap for the study of tumor cells characterized by elevated Src kinase activity.

## Competing interests

The authors declare that they have no competing interests.

## Authors' contributions

BMM performed microarray and data analyses, statistics, CEF cell culture, immunofluorescence, immuno-blotting, northern blot probe design and cloning, and contributed to experimental design. BDN derived and cultured the CNR cells and participated in experimental design. YW cultured CEF and performed northern blotting analyses. LW and NAR participated in CEF cell culture, treatment and western blotting analysis. GG participated in experimental design and coordination. PAB conceived and coordinated the study, derived the CEF, and contributed to experimental design. BMM and PAB wrote the manuscript. All authors read and approved the manuscript.

## Pre-publication history

The pre-publication history for this paper can be accessed here:

http://www.biomedcentral.com/1471-2407/10/41/prepub

## Supplementary Material

Additional file 1 Temperature regulated genes. Click here for file

Additional file 2 Pairwise comparisons of CEF infected with RCASBP(A), NY315, or SRA RSV. Click here for file

Additional file 3 Transformation-Regulated genes in CEF. Click here for file

Additional file 4 Genes regulated by ts NY72-4 infected CEF. Click here for file

Additional file 5 v-Src regulated genes identified in SR-A and ts NY72-4 RSV transformed CEF. Click here for file

Additional file 6 Genes regulated by ts NY72-4 infected CNR. Click here for file

Additional file 7 Genes commonly regulated in all three v-Src transformed cell systems. Click here for file

Additional file 8 Genes uniquely regulated in NY72-4 RSV transformed CEF and CNR. Click here for file

Additional file 9 **Expression of CD44 in normal and transformed CEF**. Surface expression of CD44 was examined by immunofluorescence in CEF infected with RCASBP(A), NY315 or SR-A RSV. NY72-4 infected CEF were either grown at the non-permissive (41.5°C) or the permissive temperature (37.5°C) for 24 hours prior to fixing and staining for CD44. Click here for file

Additional file 10 **Comparison of expression data of v-Src regulated genes as determined by gene profiling and northern blotting analyses**. Gene expression by northern blotting analysis (Figure 3) was quantified and analyzed against microarray data to confirm a correlation in gene expression as measured by the two methods. Analysis of correlation of log_2_-transformed gene expression ratios (log_2_(experimental/baseline)) indicates a strong correlation (Spearman ρ of 0.83; p < 0.0001) between northern blot and microarray gene expression estimates. This ρ value is higher than typically observed in array validations [103], and consistent with northern blotting as a superior method of gene expression validation [104]. A slope of 0.91 for the regression line indicates a nearly 1:1 ratio between log_2 _expression ratios of northern and microarray data. Click here for file

Additional file 11 **Comparison of gene expression data quantified from northern blots (Figure**3) **compared to values obtained from microarray analysis.** Click here for file

Additional file 12 **Full Pathway Express output summary**. Common pathways found to be dysregulated in the Transformation-Regulated (TR), CEF NY72-4 and CNR NY72-4 gene sets are shown. The *number of genes in the pathway *refers to the number of genes in the associated KEGG pathway. *Input genes *refer to the number of differentially expressed genes that were found in that pathway. Corrected γ p-value is a measure of significance as calculated by Pathway Express. N.D. and N.S. indicate *not determined *and *not significant *(corrected γ p-value > 0.05) respectively. Click here for file

Additional file 13 **Activation of the PI3K/PKB-Akt pathway in v-Src-transformed CEF**. The activation of PKB-Akt in RCASBP(A), NY315 and SR-A RSV infected CEF was investigated by western blotting analysis. Antibodies for PKB-Akt and the Ser-473 phosphorylated form of PKB-Akt were used to determine the expression and activation of PKB-Akt, respectively. The level of phospho-PKB-Akt was examined in cells treated with 1% DMSO (D; diluent) or the PI3K inhibitors LY290042 (LY) and wortmannin (W). PKB-Akt was hyper-phosphorylated in v-Src transformed CEF but phospho-PKB-Akt levels decreased upon treatment with the PI3K inhibitors. The activation of PKB-Akt coincided with increased Ser-9 phosphorylation of GSK3-β. Click here for file

Additional file 14 GO biological process terms most enriched in the aggressive gene signature. Click here for file
