# Supplementary material for: Cellular processes of v-Src transformation revealed by gene profiling of primary cells - Implications for human cancer
Source: BMC Cancer. 2010 Feb 12;10:41. doi: 10.1186/1471-2407-10-41 (PMC2837010; doi:10.1186/1471-2407-10-41)
Supplement: Additional file 7 — Genes commonly regulated in all three v-Src transformed cell systems. [file 1471-2407-10-41-S7.DOC]

Additional File 7 - Genes commonly regulated in all three v-Src transformed cell systems.

|  |  |  | **Linear fold change** | | |
| --- | --- | --- | --- | --- | --- |
| **Probe Set ID** | **Gene Symbol** | **Gene Title** | **SRA/RCAS** | **CEF 72-4** | **CNR 72-4** |
| Gga.817.1.S1_at | PLAU | plasminogen activator, urokinase | 44.94 | 9.78 | 6.96 |
| Gga.5778.1.S1_a_at | LOC769246 | hypothetical protein LOC769246 | 44.63 | 4.44 | 12.47 |
| Gga.666.1.S1_a_at | VIP | vasoactive intestinal peptide | 38.59 | 8.34 | 5.43 |
| Gga.11969.1.S1_at | CYTL1 | cytokine-like 1 | 20.11 | -2.64 | -2.27 |
| Gga.11456.1.S1_at | LOC418268 | similar to PLCPI=cysteine proteinase inhibitor | 18.13 | 2.13 | 12.82 |
| Gga.826.1.S1_s_at | IL8 | interleukin 8 | 14.12 | 7.41 | 4.17 |
| Gga.2680.1.S1_at | AQP1 | aquaporin 1 | 11.71 | 4.38 | 5.58 |
| GgaAffx.8298.1.S1_at | UPP1 | uridine phosphorylase 1 | 7.52 | 5.24 | 9.51 |
| Gga.150.2.S1_a_at | FLT1 | fms-related tyrosine kinase 1 (vascular endothelial growth factor/vascular permeability factor receptor) | 5.31 | 4.44 | 5.86 |
| Gga.16627.1.S1_at | LOC419930 | hypothetical LOC419930 | 5.21 | 2.51 | 10.93 |
| GgaAffx.21279.1.S1_at | UPP1 | uridine phosphorylase 1 | 5.03 | 4.17 | 8.00 |
| Gga.1111.1.S1_a_at | USP18 | ubiquitin specific peptidase 18 | 4.26 | 2.17 | 3.89 |
| Gga.12209.2.S1_a_at | LOC422672 | similar to VLLH2748 | 3.94 | 2.01 | 2.87 |
| Gga.2039.1.S1_at | HMOX1 | heme oxygenase (decycling) 1 | 3.86 | 2.33 | 6.45 |
| Gga.19230.1.S1_s_at | EAF2 | ELL associated factor 2 | 3.29 | 2.85 | 4.59 |
| Gga.4999.2.S1_at | ITGA4 | integrin, alpha 4 (antigen CD49D, alpha 4 subunit of VLA-4 receptor) | 3.16 | 2.66 | -2.36 |
| Gga.385.1.S1_at | DUSP4 | dual specificity phosphatase 4 | 3.07 | 2.93 | 4.35 |
| Gga.18801.1.S1_at | --- | Finished cDNA, clone ChEST766f10 | 3.05 | 2.23 | -2.08 |
| GgaAffx.20379.1.S1_at | --- | Finished cDNA, clone ChEST914o3 | 2.99 | 2.81 | 3.36 |
| Gga.8190.1.S1_at | LOC421544 | similar to digestive tract-specific calpain; calcium-dependent cysteine proteinase | 2.79 | 2.13 | 2.93 |
| Gga.4401.1.S1_a_at | PTGS2 | prostaglandin-endoperoxide synthase 2 (prostaglandin G/H synthase and cyclooxygenase) | 2.77 | 2.30 | 4.08 |
| Gga.19147.1.S1_at | --- | Finished cDNA, clone ChEST766h5 | 2.58 | 2.06 | 5.90 |
| Gga.1171.1.S1_at | LY6E | lymphocyte antigen 6 complex, locus E | 2.46 | 3.29 | 2.31 |
| Gga.3332.1.S1_at | MXRA8 | matrix-remodelling associated 8 | 2.23 | -2.95 | -3.03 |
| Gga.9903.1.S1_at | --- | Transcribed locus | -1.69 | -2.57 | -2.27 |
| Gga.552.1.S1_at | DIO3 | deiodinase, iodothyronine, type III | -1.80 | -5.21 | -2.58 |
| Gga.4851.1.S1_at | COL3A1 | Collagen, type III, alpha 1 (Ehlers-Danlos syndrome type IV, autosomal dominant) | -1.99 | -2.58 | -18.38 |
| Gga.16844.2.S1_at | COL3A1 | collagen, type III, alpha 1 (Ehlers-Danlos syndrome type IV, autosomal dominant) | -2.01 | -2.62 | -2.60 |
| Gga.17661.1.S1_s_at | --- | Finished cDNA, clone ChEST908h8 | -2.11 | -2.93 | -2.66 |
| Gga.17034.1.S1_s_at | YPEL2 | yippee-like 2 (Drosophila) | -2.16 | -2.45 | -2.36 |
| Gga.2839.1.S1_at | COL2A1 | collagen, type II, alpha 1 (primary osteoarthritis, spondyloepiphyseal dysplasia, congenital) | -2.19 | -2.14 | -6.45 |
| Gga.15793.1.S1_at | LOC418654 | Similar to Steryl-sulfatase precursor (Steroid sulfatase) (Steryl-sulfate sulfohydrolase) (Arylsulfatase C) (ASC) | -2.31 | -2.16 | -3.25 |
| Gga.4851.1.A1_at | COL3A1 | Collagen, type III, alpha 1 (Ehlers-Danlos syndrome type IV, autosomal dominant) | -2.33 | -2.99 | -11.31 |
| Gga.18811.1.S1_at | --- | Finished cDNA, clone ChEST987f14 | -2.41 | -4.99 | -3.48 |
| Gga.15998.1.S1_at | COLEC12 | collectin sub-family member 12 | -2.48 | -2.38 | -11.71 |
| Gga.18643.1.S1_s_at | LOC768444 | similar to voltage-gated calcium channel alpha2/delta-1 subunit | -2.48 | -2.13 | -3.84 |
| GgaAffx.25534.1.S1_s_at | COL3A1 | collagen, type III, alpha 1 (Ehlers-Danlos syndrome type IV, autosomal dominant) | -2.62 | -3.76 | -25.46 |
| Gga.17021.1.S1_at | LOC769894 | similar to Transmembrane anchor protein 1 | -3.14 | -2.97 | -4.38 |
| GgaAffx.22988.1.S1_at | --- | GgaAffx.22988.1 | -3.14 | -2.16 | -3.66 |
| Gga.16844.2.S1_s_at | COL3A1 | collagen, type III, alpha 1 (Ehlers-Danlos syndrome type IV, autosomal dominant) | -3.16 | -3.97 | -26.72 |
| GgaAffx.8024.1.S1_at | LOC771624 | hypothetical protein LOC771624 | -3.23 | -5.21 | -8.00 |
| Gga.15017.1.S1_at | LOC424815 | hypothetical LOC424815 | -3.32 | -2.11 | -2.08 |
| Gga.16392.1.S1_at | COL11A1 | collagen, type XI, alpha 1 | -3.48 | -3.03 | -13.64 |
| Gga.9052.1.S1_at | LOC768655 | similar to pim-3 protein | -3.53 | -2.66 | -5.98 |
| Gga.4941.1.S1_at | CDH11 | cadherin 11, type 2, OB-cadherin (osteoblast) | -3.63 | -2.43 | -4.38 |
| GgaAffx.20781.1.S1_at | --- | Finished cDNA, clone ChEST229d1 | -3.71 | -2.39 | -7.46 |
| Gga.2724.1.S1_at | --- | Finished cDNA, clone ChEST446j8 | -3.71 | -2.35 | -4.82 |
| GgaAffx.1064.1.S1_s_at | ATG16L1 | ATG16 autophagy related 16-like 1 (S. cerevisiae) | -4.06 | -2.10 | -3.03 |
| GgaAffx.9643.2.S1_at | PCSK5 | proprotein convertase subtilisin/kexin type 5 | -4.08 | -2.39 | -2.04 |
| GgaAffx.9643.1.S1_at | PCSK5 | proprotein convertase subtilisin/kexin type 5 | -4.26 | -2.38 | -3.68 |
| Gga.16374.1.S1_at | --- | Finished cDNA, clone ChEST84l23 | -4.38 | -2.57 | -3.39 |
| Gga.7551.1.S1_at | WISP1 | WNT1 inducible signaling pathway protein 1 | -4.56 | -2.10 | -14.32 |
| Gga.3562.1.S1_at | COL11A1 | collagen, type XI, alpha 1 | -4.82 | -3.10 | -11.71 |
| Gga.1615.1.S1_at | TMTC1 | transmembrane and tetratricopeptide repeat containing 1 | -4.82 | -2.77 | -3.23 |
| Gga.1784.1.S1_at | ITGA8 | integrin, alpha 8 | -5.03 | -3.68 | -10.34 |
| Gga.7316.1.S1_at | --- | Transcribed locus, strongly similar to XP_425758.1 similar to tumor endothelial marker 8 isoform 1 precursor; tumor endothelial marker 8; 2310008J16Rik; 2810405N18Rik [Gallus gallus] | -5.13 | -5.70 | -6.96 |
| Gga.16662.1.S1_at | --- | Gga.16662.1 | -5.21 | -2.50 | -2.25 |
| Gga.2829.1.S1_at | --- | Finished cDNA, clone ChEST888b4 | -5.35 | -2.51 | -5.10 |
| Gga.12310.1.S1_at | BBOX1 | butyrobetaine (gamma), 2-oxoglutarate dioxygenase (gamma-butyrobetaine hydroxylase) 1 | -5.78 | -2.28 | -2.33 |
| Gga.13535.1.S1_at | LOC776634 | similar to DNA-dependent protein kinase catalytic subunit | -5.78 | -2.62 | -4.53 |
| Gga.8078.1.S1_at | LOC418737 | similar to growth arrest-specific 6 | -5.90 | -6.06 | -17.63 |
| Gga.16495.1.S1_at | --- | Finished cDNA, clone ChEST400g6 | -5.94 | -2.71 | -5.70 |
| Gga.4965.3.S1_a_at | COL12A1 | collagen, type XII, alpha 1 | -6.59 | -2.68 | -3.05 |
| Gga.1784.1.S2_at | ITGA8 | integrin, alpha 8 | -6.63 | -4.96 | -8.75 |
| Gga.3573.2.S1_a_at | DKK3 | dickkopf homolog 3 (Xenopus laevis) | -7.89 | -3.14 | -4.76 |
| Gga.3972.1.S2_at | SEMA3D | sema domain, immunoglobulin domain (Ig), short basic domain, secreted, (semaphorin) 3D | -8.00 | -2.95 | -2.14 |
| Gga.17936.1.S1_at | SEMA3D | sema domain, immunoglobulin domain (Ig), short basic domain, secreted, (semaphorin) 3D | -8.17 | -2.91 | -2.62 |
| GgaAffx.26432.1.S1_s_at | COL11A1 | collagen, type XI, alpha 1 | -8.51 | -3.46 | -14.12 |
| Gga.4108.2.S1_a_at | TPM1 | tropomyosin 1 (alpha) | -8.57 | -2.03 | -3.07 |
| Gga.696.1.S1_at | BDNF | brain-derived neurotrophic factor | -9.45 | -2.53 | -4.79 |
| Gga.3972.1.S1_at | SEMA3D | sema domain, immunoglobulin domain (Ig), short basic domain, secreted, (semaphorin) 3D | -9.78 | -4.17 | -3.48 |
| Gga.665.1.S1_at | CSRP2 | cysteine and glycine-rich protein 2 | -11.00 | -3.25 | -6.54 |
| Gga.918.1.S1_at | KCNMB1 | potassium large conductance calcium-activated channel, subfamily M, beta member 1 | -11.16 | -3.68 | -5.86 |
| Gga.16710.2.S1_a_at | LOC419422 | hypothetical LOC419422 | -11.96 | -2.89 | -11.47 |
| Gga.4870.3.S1_a_at | LOC423298 | alpha-cardiac actin | -12.30 | -3.32 | -19.29 |
| Gga.2587.1.S1_at | NOV | nephroblastoma overexpressed gene | -12.73 | -5.98 | -26.72 |
| Gga.1819.1.S1_at | DIO2 | deiodinase, iodothyronine, type II | -12.82 | -5.78 | -9.85 |
| Gga.14498.1.S1_at | --- | Finished cDNA, clone ChEST533o24 | -13.09 | -3.29 | -3.01 |
| Gga.7900.2.S1_a_at | LOC426933 | similar to RIKEN cDNA 1110018M03 | -15.24 | -4.76 | -9.00 |
| Gga.4091.1.S2_a_at | MYLK | myosin, light chain kinase | -15.78 | -3.76 | -37.27 |
| Gga.3908.1.S1_at | TAGLN | Transgelin | -16.68 | -2.48 | -5.13 |
| Gga.2582.1.S1_at | TGFBI | transforming growth factor, beta-induced, 68kDa | -18.38 | -4.44 | -3.68 |
| Gga.14976.1.S1_at | --- | Finished cDNA, clone ChEST132d10 | -19.70 | -4.69 | -2.27 |
| Gga.5962.1.S1_at | --- | Gga.5962.1 | -71.51 | -6.77 | -16.00 |
