# Supplementary material for: Cellular processes of v-Src transformation revealed by gene profiling of primary cells - Implications for human cancer
Source: BMC Cancer. 2010 Feb 12;10:41. doi: 10.1186/1471-2407-10-41 (PMC2837010; doi:10.1186/1471-2407-10-41)
Supplement: Additional file 8 — Genes uniquely regulated in NY72-4 RSV transformed CEF and CNR. [file 1471-2407-10-41-S8.DOC]

Additional File 8 - Genes uniquely regulated by NY72-4 RSV in CEF and CNR cells

|  |  |  | **linear fold change** | |
| --- | --- | --- | --- | --- |
| **Probe Set ID** | **Gene Symbol** | **Gene Title** | **CNR 72-4** | **CEF 72-4** |
| Gga.14273.1.S1_at | --- | Finished cDNA, clone ChEST39a8 | 7.26 | 4.17 |
| Gga.9983.1.S1_at | LOC424161 | similar to LOC129881 protein | 6.68 | 5.46 |
| Gga.329.1.S1_at | HAS2 | Hyaluronan synthase 2 | 6.28 | 4.17 |
| Gga.4332.1.S1_at | HSPCB | heat shock 90kDa protein 1, beta | 5.46 | 3.48 |
| Gga.8920.1.S1_at | --- | Transcribed locus, weakly similar to XP_001067709.1 similar to Probable ATP-dependent RNA helicase DDX4 (DEAD box protein 4) (VASA homolog) (rVLG) [Rattus norvegicus] | 5.06 | 3.51 |
| Gga.14243.1.S1_s_at | --- | Finished cDNA, clone ChEST711g2 | 4.69 | 3.25 |
| Gga.11932.1.S1_at | UHRF1 | ubiquitin-like, containing PHD and RING finger domains, 1 | 4.66 | 2.89 |
| Gga.16057.1.S1_s_at | E2F8 | E2F transcription factor 8 | 4.63 | 2.35 |
| Gga.1334.1.S1_at | --- | Transcribed locus | 4.11 | 2.22 |
| Gga.13504.1.S1_at | RRM2 | ribonucleotide reductase M2 polypeptide | 3.92 | 2.17 |
| Gga.4514.2.S1_s_at | RCJMB04_2p9 | ribonucleotide reductase M1 polypeptide | 3.89 | 2.11 |
| Gga.17980.1.S1_at | RCJMB04_3e14 | similar to Auh protein | 3.86 | 2.62 |
| GgaAffx.11417.1.S1_at | RCJMB04_1f15 | kinesin family member 11 | 3.73 | 2.22 |
| Gga.12991.1.S1_at | --- | Finished cDNA, clone ChEST397k15 | 3.71 | 2.07 |
| GgaAffx.4223.1.S1_s_at | RCJMB04_1f15 | kinesin family member 11 | 3.43 | 2.30 |
| Gga.12108.1.S1_at | LOC418948 | similar to chromosome 13 open reading frame 3 | 3.43 | 2.07 |
| Gga.14484.1.S1_at | --- | Finished cDNA, clone ChEST321p20 | 3.41 | 2.22 |
| GgaAffx.6785.1.S1_at | LOC421505 | similar to Exonuclease 1 | 3.29 | 2.60 |
| Gga.5548.1.S1_at | LOC770430 | similar to nucleoplasmin-3 | 3.27 | 3.51 |
| Gga.4129.1.S1_at | CCNA2 | cyclin A2 | 3.23 | 2.13 |
| Gga.4340.1.S1_at | ODC1 | ornithine decarboxylase 1 | 3.14 | 2.50 |
| Gga.3551.1.S1_at | LOC395210 | osteopontin | 3.10 | 2.73 |
| GgaAffx.12448.1.S1_at | RCJMB04_15m14 | nucleolar complex associated 2 homolog (S. cerevisiae) | 3.01 | 2.31 |
| Gga.3743.1.S1_at | LOC420798 | similar to HPV16 E1 protein binding protein | 3.01 | 2.01 |
| Gga.7090.1.S1_s_at | --- | Finished cDNA, clone ChEST49g17 | 2.95 | 2.51 |
| Gga.17775.1.S1_s_at | LOC421544 | similar to digestive tract-specific calpain; calcium-dependent cysteine proteinase | 2.95 | 2.16 |
| GgaAffx.11571.1.S1_at | VRK1 | vaccinia related kinase 1 | 2.75 | 2.31 |
| Gga.16540.1.S1_s_at | HELLS | helicase, lymphoid-specific | 2.69 | 2.01 |
| GgaAffx.11513.1.S1_at | RCJMB04_2a15 | cyclin E2 | 2.68 | 2.14 |
| Gga.13515.1.S1_at | RCJMB04_11a8 | lamin B receptor | 2.66 | 2.53 |
| Gga.15350.1.S1_at | CEP55 | centrosomal protein 55kDa | 2.62 | 2.13 |
| GgaAffx.21581.1.S1_s_at | CXCL14 | chemokine (C-X-C motif) ligand 14 | 2.60 | -2.17 |
| GgaAffx.11442.1.S1_at | RCJMB04_1i4 | nuclear autoantigenic sperm protein (histone-binding) | 2.55 | 3.20 |
| Gga.10464.1.S1_at | SLC36A4 | solute carrier family 36 (proton/amino acid symporter), member 4 | 2.55 | 3.07 |
| GgaAffx.12549.1.S1_s_at | LOC768335 /// RCJMB04_17m23 | coiled-coil domain containing 13 /// similar to similar to hypothetical protein FLJ25467 | 2.48 | 2.43 |
| GgaAffx.20419.1.S1_at | --- | Finished cDNA, clone ChEST761a8 | 2.48 | 2.01 |
| Gga.16406.1.S1_at | --- | Finished cDNA, clone ChEST237g13 | 2.45 | 2.38 |
| Gga.5809.1.S1_at | TTC35 | tetratricopeptide repeat domain 35 | 2.41 | 2.23 |
| GgaAffx.6598.1.S1_at | LOC425049 | similar to Tubulin alpha-3/alpha-7 chain (Alpha-tubulin 3/7) (Alpha-tubulin isotype M-alpha-3/7) | 2.41 | 2.11 |
| GgaAffx.23188.1.A1_s_at | LOC770430 | similar to nucleoplasmin-3 | 2.39 | 2.57 |
| Gga.2067.1.S1_at | RCJMB04_9a7 | kinesin heavy chain member 2A | 2.39 | 2.46 |
| GgaAffx.12498.1.S1_s_at | RCJMB04_16n14 | similar to LOC330189 protein | 2.35 | 3.63 |
| Gga.4074.1.S1_s_at | LOC420709 | similar to RIKEN cDNA 1110059G10 | 2.35 | 2.38 |
| Gga.7431.1.S1_at | LOC424307 | hypothetical LOC424307 | 2.35 | 2.04 |
| Gga.13253.1.S1_at | LOC421074 | similar to sudD suppressor of bimD6 homolog | 2.33 | 2.57 |
| GgaAffx.6936.1.S1_s_at | RCJMB04_19b7 | ubiquitin specific peptidase 1 | 2.31 | 2.20 |
| Gga.7042.3.A1_a_at | --- | --- | 2.31 | 2.04 |
| GgaAffx.21456.1.S1_at | LOC422697 | Hypothetical LOC422697 | 2.30 | 2.53 |
| GgaAffx.12597.1.S1_at | RCJMB04_19b7 | ubiquitin specific peptidase 1 | 2.30 | 2.04 |
| Gga.13284.1.S1_at | --- | Finished cDNA, clone ChEST975n18 | 2.27 | 2.31 |
| Gga.2476.1.S1_at | HAT1 | histone acetyltransferase 1 | 2.14 | 2.04 |
| GgaAffx.3414.1.S1_at | RCJMB04_10p19 | zinc finger, DHHC-type containing 21 | 2.13 | 2.31 |
| Gga.13466.1.S1_at | HNRPH3 | heterogeneous nuclear ribonucleoprotein H3 (2H9) | 2.13 | 2.06 |
| Gga.7238.1.S1_s_at | RCJMB04_19j12 | oxoglutarate (alpha-ketoglutarate) dehydrogenase (lipoamide) | 2.13 | 2.03 |
| Gga.9212.1.S1_at | LOC422880 | similar to RIKEN cDNA 2610033H07 gene | 2.10 | 2.73 |
| GgaAffx.11645.1.S1_at | RCJMB04_3d14 | septin 6 | 2.10 | 2.08 |
| Gga.9212.1.S1_s_at | LOC422880 | similar to RIKEN cDNA 2610033H07 gene | 2.06 | 2.45 |
| Gga.19048.1.S1_at | ATAD3B | ATPase family, AAA domain containing 3B | 2.06 | 2.04 |
| Gga.8541.1.S1_at | TNKS | tankyrase, TRF1-interacting ankyrin-related ADP-ribose polymerase | 2.04 | 2.45 |
| GgaAffx.20470.1.S1_at | --- | Finished cDNA, clone ChEST646l7 | 2.04 | 2.20 |
| Gga.4942.1.S1_at | HSP70 | heat shock protein 70 | -2.08 | -2.51 |
| Gga.1132.1.S1_at | ME1 | malic enzyme 1, NADP(+)-dependent, cytosolic | -2.10 | -2.14 |
| GgaAffx.23017.1.S1_at | --- | --- | -2.13 | -2.30 |
| Gga.14155.1.S1_at | TSPAN2 | tetraspanin 2 | -2.16 | -2.03 |
| GgaAffx.21171.1.S1_s_at | BTG2 | BTG family, member 2 | -2.17 | -2.07 |
| Gga.20.1.S2_at | --- | --- | -2.17 | -3.14 |
| Gga.4663.1.S1_at | PSPH | phosphoserine phosphatase | -2.20 | -2.50 |
| Gga.6213.1.S1_at | --- | Finished cDNA, clone ChEST296l24 | -2.25 | -2.27 |
| Gga.4168.1.S1_at | SERPINH1 | serpin peptidase inhibitor, clade H (heat shock protein 47), member 1, (collagen binding protein 1) | -2.28 | -2.89 |
| Gga.4588.1.S1_at | CDKN1B | Cyclin-dependent kinase inhibitor 1B (p27, Kip1) | -2.41 | -2.08 |
| GgaAffx.20596.1.S1_at | --- | Finished cDNA, clone ChEST698h14 | -2.46 | -2.08 |
| Gga.8904.1.S1_at | --- | Transcribed locus | -2.48 | -3.66 |
| GgaAffx.11918.1.S1_s_at | MXD4 | MAX dimerization protein 4 | -2.58 | -3.48 |
| Gga.10110.1.S1_at | --- | Finished cDNA, clone ChEST992o1 | -2.97 | -2.14 |
| Gga.9317.1.S1_at | --- | Transcribed locus | -2.99 | -2.00 |
| GgaAffx.4585.1.S1_at | LOC422293 | hypothetical LOC422293 | -3.12 | -2.25 |
| Gga.16921.1.S1_at | --- | --- | -3.34 | -2.83 |
| GgaAffx.10088.2.A1_s_at | LOC421839 | similar to putative serine protease 35 | -3.56 | -3.48 |
| GgaAffx.13119.1.S1_s_at | RCJMB04_32i2 | ribosomal protein S14 | -3.92 | -2.38 |
| Gga.13838.1.S1_at | --- | Finished cDNA, clone ChEST64i7 | -4.14 | -5.58 |
| Gga.13235.1.S1_at | --- | Finished cDNA, clone ChEST56g13 | -4.29 | -2.39 |
| Gga.5933.1.S1_at | --- | Transcribed locus | -4.53 | -2.01 |
| GgaAffx.21413.1.S1_s_at | --- | Finished cDNA, clone ChEST629c13 | -4.99 | -4.17 |
| GgaAffx.23826.7.S1_s_at | RCJMB04_18a15 | PDZ and LIM domain 5 | -5.06 | -2.11 |
| Gga.4724.1.S2_at | HSP90B1 | heat shock protein 90kDa beta (Grp94), member 1 | -5.21 | -3.05 |
| GgaAffx.10088.1.S1_at | LOC421839 | similar to putative serine protease 35 | -5.46 | -4.11 |
| Gga.1663.1.S1_s_at | LHFP | lipoma HMGIC fusion partner | -8.11 | -2.68 |
| Gga.3109.1.A1_s_at | RCJMB04_2f9 | selenoprotein P, plasma, 1 | -8.94 | -3.12 |
| GgaAffx.11541.1.S1_at | RCJMB04_2f9 | selenoprotein P, plasma, 1 | -10.78 | -3.34 |
| Gga.837.1.S1_a_at | NPY | neuropeptide Y | -20.39 | -3.86 |
| Gga.4536.2.S1_a_at | AKR1D1 | aldo-keto reductase family 1, member D1 (delta 4-3-ketosteroid-5-beta-reductase) | -20.82 | -2.10 |
