# Supplementary figures and images for: Cellular processes of v-Src transformation revealed by gene profiling of primary cells - Implications for human cancer
Source: BMC Cancer. 2010 Feb 12;10:41. doi: 10.1186/1471-2407-10-41 (PMC2837010; doi:10.1186/1471-2407-10-41)

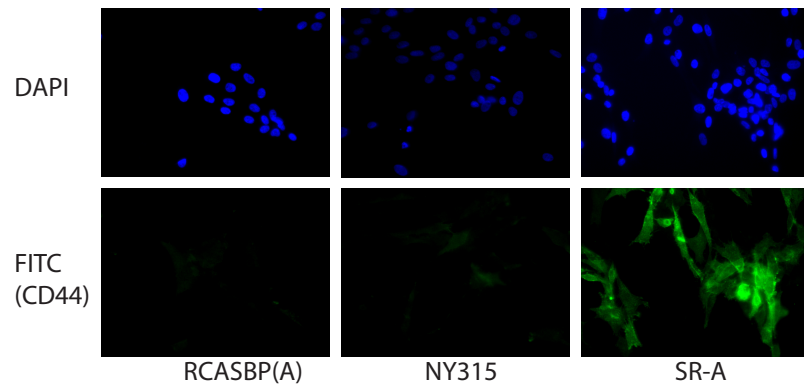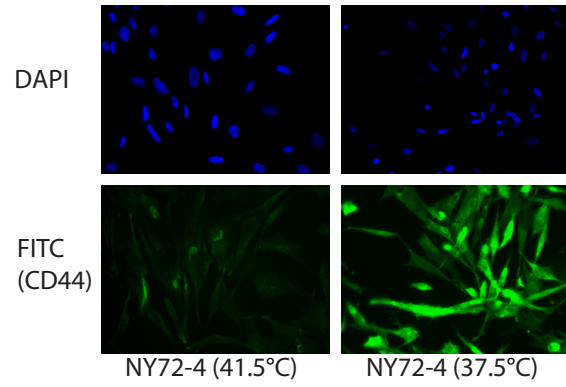

Supplement: Additional file 9 — Expression of CD44 in normal and transformed CEF. Surface expression of CD44 was examined by immunofluorescence in CEF infected with RCASBP(A), NY315 or SR-A RSV. NY72-4 infected CEF were either grown at the non-permissive (41.5°C) or the permissive temperature (37.5°C) for 24 hours prior to fixing and staining for CD44. [file 1471-2407-10-41-S9.PDF]

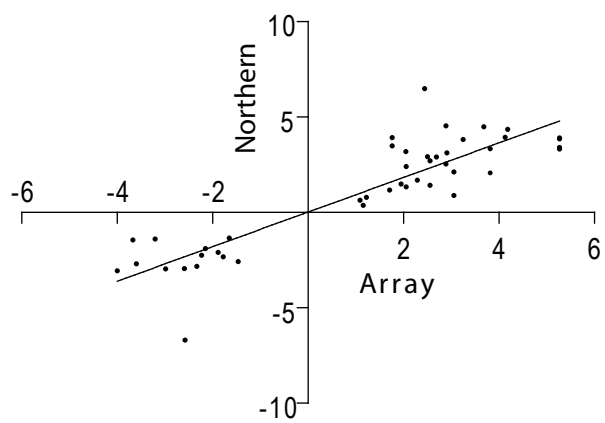

Supplement: Additional file 10 — Comparison of expression data of v-Src regulated genes as determined by gene profiling and northern blotting analyses. Gene expression by northern blotting analysis (Figure 3) was quantified and analyzed against microarray data to confirm a correlation in gene expression as measured by the two methods. Analysis of correlation of log2-transformed gene expression ratios (log2(experimental/baseline)) indicates a strong correlation (Spearman ρ of 0.83; p < 0.0001) between northern blot and microarray gene expression estimates. This ρ value is higher than typically observed in array validations [103], and consistent with northern blotting as a superior method of gene expression validation [104]. A slope of 0.91 for the regression line indicates a nearly 1:1 ratio between log2 expression ratios of northern and microarray data. [file 1471-2407-10-41-S10.PDF]

RCASBP(A)

NY315 RSV

SR-A RSV

D

LY

W

D

LY

W

D

LY

W

treatment

 $\alpha$ -PKB $\alpha$ -pPKB $\alpha$ -pGSK3 $\beta$  $\alpha$ -Erk

1

2

3

4

5

6

7

8

9

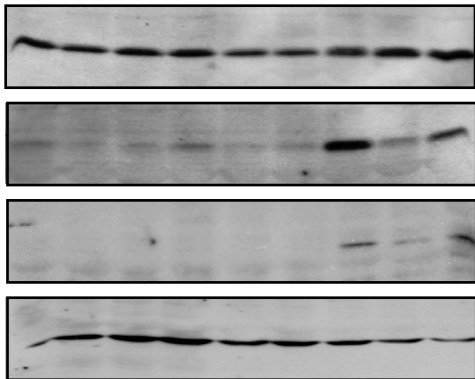

Supplement: Additional file 13 — Activation of the PI3K/PKB-Akt pathway in v-Src-transformed CEF. The activation of PKB-Akt in RCASBP(A), NY315 and SR-A RSV infected CEF was investigated by western blotting analysis. Antibodies for PKB-Akt and the Ser-473 phosphorylated form of PKB-Akt were used to determine the expression and activation of PKB-Akt, respectively. The level of phospho-PKB-Akt was examined in cells treated with 1% DMSO (D; diluent) or the PI3K inhibitors LY290042 (LY) and wortmannin (W). PKB-Akt was hyper-phosphorylated in v-Src transformed CEF but phospho-PKB-Akt levels decreased upon treatment with the PI3K inhibitors. The activation of PKB-Akt coincided with increased Ser-9 phosphorylation of GSK3-β. [file 1471-2407-10-41-S13.PDF]
