# Supplementary material for: Cellular processes of v-Src transformation revealed by gene profiling of primary cells - Implications for human cancer
Source: BMC Cancer. 2010 Feb 12;10:41. doi: 10.1186/1471-2407-10-41 (PMC2837010; doi:10.1186/1471-2407-10-41)
Supplement: Additional file 11 — Comparison of gene expression data quantified from northern blots (Figure3) compared to values obtained from microarray analysis. [file 1471-2407-10-41-S11.DOC]

**Additional File 11 - Comparison of gene expression data quantified from northern blots (Figure 3) compared to values obtained from microarray analysis.**

|  | Northern blot gene expression fold change | | | | | Microarray gene expression fold change | | | | |
| --- | --- | --- | --- | --- | --- | --- | --- | --- | --- | --- |
| Gene | CEF SRA/RCAS | CEF SRA/NY315 | CEF NY315/RCAS | CEF NY72-4P/CEF NY72-4NP | CNR NY72-4P/CNR NY72-4NP | CEF SRA/RCAS | CEF SRA/NY315 | CEF NY315/RCAS | CEF NY72-4P/CEF NY72-4NP | CNR NY72-4P/CNR NY72-4NP |
| CD44 | 11.27 | 9.14 | 1.23 | 3.09 | N.D. | 3.39 | 4.14 | N.S. | N.S. | 3.73 |
| DKK3 | -7.75 | -7.66 | -1.01 | -2.54 | N.D. | -7.89 | -6.02 | N.S. | -3.14 | -4.76 |
| HMOX1 | 2.83 | 1.29 | 2.19 | 1.73 | 7.51 | 3.86 | 2.23 | N.S. | 2.33 | 6.45 |
| IL8^1^ | 4.22 | 2.69 | 1.57 | 23.31 | N.D. | 14.12 | 5.90 | N.S. | 7.41 | 4.17 |
| IL8^2^ | 10.16 | 6.54 | 1.55 | 5.82 | 5.33 | 14.12 | 5.90 | N.S. | 7.41 | 4.17 |
| ITGA1 | -4.73 | -5.02 | 1.06 | -20.80 | N.D. | -4.69 | -3.43 | N.S. | N.S. | -3.63 |
| ITGA6 | 15.25 | 7.61 | 2.00 | 2.26 | N.D. | 3.39 | 5.66 | N.S. | 3.27 | N.S. |
| ITGA8 | -7.11 | -5.93 | -1.20 | -4.24 | N.D. | -5.03 | -2.75 | N.S. | -3.68 | -8.75 |
| NOV | -2.72 | -2.63 | -1.03 | -103.38 | N.D. | -12.73 | -9.19 | N.S. | -5.98 | -26.72 |
| PLCPI | 20.59 | 15.44 | 1.33 | 1.56 | 22.60 | 18.13 | 17.51 | N.S. | 2.13 | 12.82 |
| THBS2 | -8.35 | -6.45 | -1.29 | -3.72 | N.D. | -16.00 | -12.13 | N.S. | -4.44 | N.S. |
| UPP1 | 8.71 | 3.22 | 2.70 | 2.55 | 14.18 | 7.52 | 4.89 | N.S. | 4.17 | 9.51 |
| VIP^1^ | 10.62 | 10.02 | 1.06 | 1.85 | N.D. | 38.59 | 38.59 | N.S. | 8.34 | 5.43 |
| VIP^2^ | 15.00 | 14.48 | 1.04 | 4.35 | 89.91 | 38.59 | 38.59 | N.S. | 8.34 | 5.43 |

N.D. No data

N.S. Not statistically significant

1. Figure 3A

2. Figure 3C
