# Supplementary material for: Cellular processes of v-Src transformation revealed by gene profiling of primary cells - Implications for human cancer
Source: BMC Cancer. 2010 Feb 12;10:41. doi: 10.1186/1471-2407-10-41 (PMC2837010; doi:10.1186/1471-2407-10-41)
Supplement: Additional file 12 — Full Pathway Express output summary. Common pathways found to be dysregulated in the Transformation-Regulated (TR), CEF NY72-4 and CNR NY72-4 gene sets are shown. The number of genes in the pathway refers to the number of genes in the associated KEGG pathway. Input genes refer to the number of differentially expressed genes that were found in that pathway. Corrected γ p-value is a measure of significance as calculated by Pathway Express. N.D. and N.S. indicate not determined and not significant (corrected γ p-value > 0.05) respectively. [file 1471-2407-10-41-S12.DOC]

**Additional File 12 -** **Full** **Pathway Express output summary.**

|  |  | #Input genes in pathway | | | Corrected γ p-value | | |
| --- | --- | --- | --- | --- | --- | --- | --- |
| Pathway Name | genes in pathway | TR | CEF NY72-4 | CNR NY72-4 | TR | CEF NY72-4 | CNR NY72-4 |
| ECM-receptor interaction | 87 | 19 | 10 | 14 | 1.83E-11 | 3.37E-06 | 3.14E-07 |
| Focal adhesion | 195 | 26 | 14 | 19 | 6.87E-11 | 1.44E-06 | 3.47E-06 |
| Leukocyte transendothelial migration | 116 | 7 | 2 | 7 | 0.003187 | 0.017336 | 0.027548 |
| Phosphatidylinositol signaling system | 77 | 3 | 1 | 3 | 7.71E-10 | 4.8E-11 | 0.001714 |
| Small cell lung cancer | 86 | 12 | 6 | 8 | 2.56E-05 | 0.009194 | 0.012553 |
| Complement and coagulation cascades | 69 | 4 | 2 | N.D. | 0.001305 | 0.021465 | N.S. |
| Epithelial cell signaling in Helicobacter pylori infection | 67 | 3 | 1 | N.D. | 0.002804 | 1.09E-05 | N.S. |
| Regulation of actin cytoskeleton | 208 | 19 | N.D. | 11 | 1.27E-05 | N.S. | 0.005032 |
| TGF-beta signaling pathway | 84 | 10 | 7 | N.D. | 2.9E-06 | 0.000212 | N.S. |
| Type II diabetes mellitus | 44 | 4 | 1 | N.D. | 0.025986 | 0.02847 | N.S. |
| Adipocytokine signaling pathway | 72 | N.D. | N.D. | 1 | N.S. | N.S. | 0.028639 |
| Apoptosis | 84 | 6 | N.D. | N.D. | 0.039495 | N.S. | N.S. |
| Axon guidance | 128 | N.D. | N.D. | 8 | N.S. | N.S. | 0.038615 |
| B cell receptor signaling pathway | 63 | 6 | N.D. | N.D. | 0.014251 | N.S. | N.S. |
| Calcium signaling pathway | 175 | N.D. | N.D. | 9 | N.S. | N.S. | 0.036604 |
| Cell adhesion molecules (CAMs) | 132 | 5 | N.D. | N.D. | 8.42E-38 | N.S. | N.S. |
| Cell cycle | 114 | N.D. | N.D. | 18 | N.S. | N.S. | 5.74E-08 |
| Chronic myeloid leukemia | 76 | 6 | N.D. | N.D. | 0.043935 | N.S. | N.S. |
| Colorectal cancer | 85 | 8 | N.D. | N.D. | 0.005792 | N.S. | N.S. |
| Cytokine-cytokine receptor interaction | 256 | 13 | N.D. | N.D. | 0.022571 | N.S. | N.S. |
| Glioma | 64 | 5 | N.D. | N.D. | 0.018471 | N.S. | N.S. |
| Jak-STAT signaling pathway | 153 | 10 | N.D. | N.D. | 0.015333 | N.S. | N.S. |
| MAPK signaling pathway | 256 | 16 | N.D. | N.D. | 0.002903 | N.S. | N.S. |
| Maturity onset diabetes of the young | 25 | N.D. | 2 | N.D. | N.S. | 0.048891 | N.S. |
| Melanogenesis | 102 | N.D. | N.D. | 5 | N.S. | N.S. | 0.019509 |
| Melanoma | 71 | 6 | N.D. | N.D. | 0.017322 | N.S. | N.S. |
| Non-small cell lung cancer | 53 | 5 | N.D. | N.D. | 0.042782 | N.S. | N.S. |
| Pancreatic cancer | 73 | 7 | N.D. | N.D. | 0.005432 | N.S. | N.S. |
| Renal cell carcinoma | 69 | N.D. | N.D. | 6 | N.S. | N.S. | 0.034607 |
| T cell receptor signaling pathway | 93 | 6 | N.D. | N.D. | 0.040059 | N.S. | N.S. |
| Tight junction | 119 | 8 | N.D. | N.D. | 0.025269 | N.S. | N.S. |
| Toll-like receptor signaling pathway | 90 | 8 | N.D. | N.D. | 0.004474 | N.S. | N.S. |

Common pathways found to be dysregulated in the Transformation-Regulated (TR), CEF NY72-4 and CNR NY72-4 gene sets are shown. The *number of genes in the pathway* refers to the number of genes in the associated KEGG pathway. *Input genes* refer to the number of differentially expressed genes that were found in that pathway. Corrected γ p-value is a measure of significance as calculated by Pathway Express. N.D. and N.S. indicate *not determined* and *not significant* (corrected γ p-value > 0.05) respectively.
