# Supplementary material for: Cellular processes of v-Src transformation revealed by gene profiling of primary cells - Implications for human cancer
Source: BMC Cancer. 2010 Feb 12;10:41. doi: 10.1186/1471-2407-10-41 (PMC2837010; doi:10.1186/1471-2407-10-41)
Supplement: Additional file 14 — GO biological process terms most enriched in the aggressive gene signature. [file 1471-2407-10-41-S14.DOC]

**Additional File 14** - **GO biological process terms most enriched in the aggressive gene signature.**

| **GO ID** | **GOBP Term** | **PValue** | **Genes** | **Fold Enrichment** |
| --- | --- | --- | --- | --- |
| 7049 | cell cycle | 6.12E-05 | KIF11, EXO1, IL8, UHRF1, HELLS, CCNE2, CEP55, NASP, E2F8, CCNA2, | 5.21 |
| 51301 | cell division | 1.60E-03 | KIF11, HELLS, CCNE2, CEP55, CCNA2, | 9.50 |
| 6259 | DNA metabolic process | 1.65E-03 | EXO1, UHRF1, HELLS, CCNE2, NASP, RRM2, USP1, RRM1, | 4.33 |
| 6260 | DNA replication | 1.72E-03 | EXO1, CCNE2, NASP, RRM2, RRM1, | 9.31 |
| 279 | M phase | 2.84E-03 | KIF11, EXO1, HELLS, CEP55, CCNA2, | 8.11 |
| 22402 | cell cycle process | 4.02E-03 | KIF11, EXO1, IL8, HELLS, CCNE2, CEP55, CCNA2, | 4.35 |
| 6974 | response to DNA damage stimulus | 4.39E-03 | EXO1, UHRF1, USP1, HMOX1, CCNA2, | 7.18 |
| 6950 | response to stress | 5.95E-03 | EXO1, PLAU, IL8, UHRF1, HSP90AB1, USP1, HMOX1, CCNA2, | 3.44 |
| 22403 | cell cycle phase | 6.11E-03 | KIF11, EXO1, HELLS, CEP55, CCNA2, | 6.54 |
| 9719 | response to endogenous stimulus | 9.39E-03 | EXO1, UHRF1, USP1, HMOX1, CCNA2, | 5.77 |
| 7067 | mitosis | 1.15E-02 | KIF11, HELLS, CEP55, CCNA2, | 8.20 |
| 87 | M phase of mitotic cell cycle | 1.18E-02 | KIF11, HELLS, CEP55, CCNA2, | 8.13 |
| 8283 | cell proliferation | 2.32E-02 | IL8, UHRF1, HELLS, ODC1, NASP, HMOX1, | 3.51 |
| 278 | mitotic cell cycle | 2.80E-02 | KIF11, HELLS, CEP55, CCNA2, | 5.85 |
| 42221 | response to chemical stimulus | 3.60E-02 | PLAU, IL8, HSP90AB1, PDCD6, HMOX1, | 3.84 |
| 48514 | blood vessel morphogenesis | 4.66E-02 | IL8, HMOX1, ITGA4, | 8.41 |
